# Establishing RTS,S/AS01 as a benchmark for comparison to next-generation malaria vaccines in a mouse model

**DOI:** 10.1038/s41541-024-00819-x

**Published:** 2024-02-10

**Authors:** Emily Locke, Yevel Flores-Garcia, Bryan T. Mayer, Randall S. MacGill, Bhavesh Borate, Berenice Salgado-Jimenez, Monica W. Gerber, Shamika Mathis-Torres, Sarah Shapiro, C. Richter King, Fidel Zavala

**Affiliations:** 1 Center for Vaccine Innovation and Access, PATH, Washington, DC 20001 USA; 2 Department of Molecular Microbiology and Immunology, Malaria Research Institute, Johns Hopkins Bloomberg School of Public Health, Baltimore, MD USA; 3 Vaccine and Infectious Disease Division, Fred Hutchison Cancer Research Center, Seattle, WA 98109 USA

**Keywords:** Malaria, Infectious diseases

## Abstract

New strategies are needed to reduce the incidence of malaria, and promising approaches include vaccines targeting the circumsporozoite protein (CSP). To improve upon the malaria vaccine, RTS,S/AS01, it is essential to standardize preclinical assays to measure the potency of next-generation vaccines against this benchmark. We focus on RTS,S/AS01-induced antibody responses and functional activity in conjunction with robust statistical analyses. Transgenic *Plasmodium berghei* sporozoites containing full-length *P. falciparum* CSP (tg*Pb-Pf*CSP) allow two assessments of efficacy: quantitative reduction in liver infection following intravenous challenge, and sterile protection from mosquito bite challenge. Two or three doses of RTS,S/AS01 were given intramuscularly at 3-week intervals, with challenge 2-weeks after the last vaccination. Minimal inter- and intra-assay variability indicates the reproducibility of the methods. Importantly, the range of this model is suitable for screening more potent vaccines. Levels of induced anti-CSP antibody 2A10 equivalency were also associated with activity: 105 μg/mL (95% CI: 68.8, 141) reduced liver infection by 50%, whereas 285 μg/mL (95% CI: 166, 404) is required for 50% sterile protection from mosquito bite challenge. Additionally, the liver burden model was able to differentiate between protected and non-protected human plasma samples from a controlled human malaria infection study, supporting these models’ relevance and predictive capability. Comparison in animal models of CSP-based vaccine candidates to RTS,S/AS01 is now possible under well controlled conditions. Assessment of the quality of induced antibodies, likely a determinant of durability of protection in humans, should be possible using these methods.

## Introduction

Despite significant progress in reducing the morbidity from malaria, recent data indicate that this momentum has stalled and possibly even reversed in some high burden countries^1^. Malaria deaths, mostly due to *Plasmodium falciparum*, decreased from 896,000 in 2000–562,000 in 2015. Unfortunately, in 2020, malaria deaths rose by 12% to 631,000, dropping slightly to 608,000 in 2022^1^. The World Health Organization (WHO) and the European Medicines Agency (EMA) recommended widespread use of RTS,S/AS01, among children in sub-Saharan Africa and in other regions with moderate to high *P. falciparum* malaria transmission. This was based on extensive Phase 2 and 3 testing and results from a pilot implementation program in more than 900,000 children in Ghana, Kenya, and Malawi^2–4^. In October 2023, WHO recommended R21/Matrix-M for children living in endemic areas^1^. However, there remains an urgent need for additional tools, including superior vaccines, to combat this disease.

RTS,S/AS01, under development since 1987^5–7^, targets the circumsporozoite protein (CSP), the major protein on the surface of infectious sporozoites^7^. The vaccine is well tolerated and has shown modest efficacy against clinical disease in young children, however efficacy wanes over time^8^. Antibodies against the NPNA major repeat region are associated with efficacy and durability of protective responses, as the decline of both anti-NPNA antibody titer and efficacy follows a biphasic pattern of decay in which an initial rapid drop in anti-NPNA antibody titers and efficacy is followed by a slower prolonged period of decline, which is typical of many vaccine-induced responses^9^. Seasonal vaccination with RTS,S/AS01 and administration of seasonal malaria chemoprevention (SMC) were recently evaluated alone and in combination in five to 17 month old children in Mali and Burkina Faso. RTS,S/AS01 was found to be noninferior to SMC in preventing uncomplicated malaria. When combined, the two interventions reduced the incidence of uncomplicated malaria, severe malaria, and death from malaria more than either intervention alone^2^. Another *Pf*CSP-based vaccine under development, R21/Matrix-M, recently recommended by WHO, was evaluated recently via a seasonal vaccination strategy in a Phase 2b study in 5- to 17-month-old children in Burkina Faso^10^. Efficacy over the transmission season of ~6 months appears promising, and high-level efficacy can be extended by additional booster doses given just before the rainy season. Overall, the efficacy was comparable to short-term efficacy demonstrated by RTS,S/AS01 in similar settings^11^. Follow-on studies with R21/Matrix-M in larger populations in different transmission settings will be conducted to gain further information on vaccine efficacy and longevity of responses^12^. These two vaccines validate the CSP target for vaccination and encourage the design of next-generation vaccines that improve upon the durability of protective efficacy. Passive vaccination using monoclonal antibodies (mAbs) that bind to CSP are also showing promise in clinical trials and are currently being assessed in the target population of young African children^13–17^.

Mouse models of *P. falciparum* have been developed for preclinical testing of vaccines and mAbs that target the CSP protein using a transgenic *P. berghei* sporozoite which contains full-length *P.falciparum* CSP, tg*Pb-Pf*CSP^18,19^. The model allows two assessments of efficacy: first, reduction in liver infection following intravenous (IV) sporozoite challenge, and second, sterile protection following mosquito bite challenge. Mice can be immunized via either passive transfer of antibodies^20^ or active immunization with test vaccines, the focus of this study. The reduction in parasite liver infection enables a quantitative assessment of the ability of antibodies to inhibit liver infection. This approach allows a consistent number of sporozoites to be injected into each mouse, enabling a quantitative measurement of the effect of antibody inhibition. Alternatively, sterile protection following challenge by infected mosquitoes and prevention of progression to blood-stage infection can be used as a more clinically relevant readout; however, as previously reported, more variability is observed likely because the number of sporozoites introduced into a host depends on factors such as mosquito size, time since last feed, and feeding area on host^21,22^.

In a previous study, we rigorously characterized the liver infection and protection from mosquito bite models for inter- and intra-experiment reproducibility using two highly potent anti-CSP mAbs. We found the models are suitable for measuring mAb potency consistently and providing guidance for selecting potent mAbs for further development^20^. This approach has since been validated with the progression of CIS43LS, L9LS, and MAM01 mAbs which have advanced to clinical testing following down-selection and confirmation of activity in similar models^16,17,23–26^. As a follow-on effort, we are now interrogating these models for their suitability for providing guidance for the selection of next-generation CSP-based vaccines.

Other investigators have used similar but not identical mouse models to draw conclusions on relative potency from intra-study comparisons of vaccines^27,28^ and mAbs^29,30^. In addition, recently RTS,S/AS01 has been used in a similar mouse model to explore antigen and vaccine titration using sterile protection as an endpoint^31^. In the present study, we focus on a clinically relevant benchmark for comparison studies by studying responses elicited by RTS,S/AS01 immunization. Our goal was to enable preclinical comparisons of next-generation *Pf*CSP-targeted vaccine candidates now under development. We focus on the reproducibility of responses as measured by antibody level and functional activity in conjunction with robust statistical analyses. We also describe an approach to measuring antibody quality or potency as a key feature of a desired next-generation vaccine.

## Results

As with our previous studies on mAbs, we sought to determine if mouse challenge studies could be conducted with appropriate inter- and intra-assay variability such that test vaccines could be compared to an RTS,S/AS01 benchmark.

### Reduction in liver infection (parasite liver burden): Assay consistency

Five independent experiments were conducted using a 200 fold range of RTS,S (0.05 μg–10 μg) in a constant amount of adjuvant (10-fold diluted original AS01_E_ adjuvant dose, representing 2.5 µg of the TLR4 ligand 3-*O*-desacyl-4’-monophosphoryl lipid A (MPL), and 2.5 µg of the QS-21 saponin) (Table 1). The fixed amount of adjuvant allows comparison of immunogens as a single experimental variable. Two vaccine administrations were tested in three experiments and three administrations in two experiments. Immunizations were given intramuscularly (IM) at 3-week intervals, with IV challenge using 2,000 tg*Pb-Pf*CSP sporozoites 2-weeks after the last vaccination. These sporozoites also carry the gene for luciferase and, thus, liver infection can be monitored as previously described^18^ by bioluminescence (Flux) using the IVIS in vivo imaging system. A negative control consisted of mice receiving only PBS prior to challenge and positive control mice received 300 μg of the potent major CSP repeat-specific (NPNA_6_) AB317^32–34^ via IV passive transfer 16 h prior to challenge.

**Table 1:** Experimental design of five reduction in liver infection experiments showing group sizes

Experiment ID	Administrations (in vaccine groups)	Control groups (# mice/group)			Vaccine groups (# mice/group)				
		AB317	Naïve (No vaccine)	Background (d-luciferin)	RTS,S dose levels				
					0.05 μg	0.2 μg	1 μg	5 μg	10 μg
2x Trial 1	2	5	5	2	5	5	5	5	5
2x Trial 2	2	5	5	2	5	5	5	5	5
2x Trial 3	2	5	5	2	5	5	5	5	5
3x Trial 1	3	5	5	2	5	5	5	5	5
3x Trial 2	3	5	5	2	5	5	5	5	5

Different doses of RTS,S in a constant amount of AS01 adjuvant were administered intramuscularly at 3-week intervals, with IV challenge from 2000 tg*Pb-Pf*CSP sporozoites 2-weeks after the last vaccination. For the AB317 groups, only one administration was given 16 h prior to challenge.

The overall dynamic range of the assay was determined by an upper limit set by log_10_ flux of the unvaccinated (naïve) infected mice (log_10_ flux mean 7.20 ± 0.149 standard deviation [SD]) and the lower limit set by the log_10_ flux of mice that were not challenged but injected with the luciferase substrate D-luciferin (log_10_ flux mean 5.37 ± 0.084 SD) (Table 2) as a measure of assay background. Control measurements and the overall dynamic range (1.8 log_10_ flux) were consistent with results previously reported^20^, which reflects well on the consistency of the assay. In the present and previous studies, there was also nearly complete inhibition of liver infection by passive administration of AB317 (mean log_10_ flux 5.48 ± 0.130 SD)^20^.

**Table 2:** Flux variation estimated using linear mixed effects models for the pooled control groups, assay background measurements, and pooled vaccine groups (each total dose and dose-level combination)

Treatment	Number of admin.	Treatment group	Animals per group (N)		Mean log_10_flux (photons/sec)	SD (log_10_ flux)		
			Mice	Experiments		Between- experiment	Within- experiment	Intraclass correlation (%)
**Control**		Naïve infected	25	5	7.20	0.077	0.131	25.7
		AB317	25	5	5.48	0.000	0.130	0.0
		Assay background	12	5	5.37	0.000	0.084	0.0
**Vaccine**	**2**	0.05 µg RTS,S	15	3	7.21	0.100	0.146	31.8
		0.2 µg RTS,S	15	3	6.92	0.059	0.160	11.8
		1 µg RTS,S	15	3	6.68	0.000	0.276	0.0
		5 µg RTS,S	15	3	6.51	0.091	0.221	14.4
		10 µg RTS,S	15	3	6.67	0.000	0.221	0.0
	**3**	0.05 µg RTS,S	10	2	6.72	0.122	0.390	8.9
		0.2 µg RTS,S	10	2	6.29	0.000	0.382	0.0
		1 µg RTS,S	10	2	5.91	0.316	0.510	27.7
		5 µg RTS,S	10	2	5.89	0.000	0.341	0.0
		10 µg RTS,S	10	2	5.76	0.000	0.355	0.0

The mean log_10_ flux (photons/second) is the mean across experiments (the fixed effect intercept) estimated from the model. The SD log_10_ flux is shown as between (inter-) and within (intra-) experiment. The intraclass correlation is the proportion of the total variance attributed to differences between experiments; a value of zero implies concordance across experiments.

Analysis of the pooled results from the control groups in all five experiments showed there was excellent agreement in flux measurements between experiments (Table 2) with assay variation largely attributable to intra-assay variance. For mice in the negative control group or receiving AB317, there was essentially no estimated inter-assay variation. As noted previously, this low level of variability suggests the sporozoites were prepared and handled consistently to maintain uniformity in load and infectiousness across batches.

In RTS,S/AS01 vaccination experiments, consistency of reduction in liver infection was also seen at each dose level and immunization regimen as indicated by the low SD within and between experiments (Table 2, Supplementary Fig. 1). At each administered dose level there was variation between individual mice as expected. There was some increased intra-assay variation observed for the groups immunized with RTS,S/AS01 compared to the variation in control groups, with maximum SD of 0.51 log_10_ flux, ~3 fold from the mean flux (Table 2, Supplementary Fig. 1). Intra-assay SDs ranged from 0.084–0.131, or 1.21 fold–1.35 fold, across all control groups and 0.146–0.510 (1.4 fold–3.24 fold) in treatment groups (Table 2, Supplementary Fig. 1). These SDs were generally consistent with SDs observed previously with anti-NPNA mAb AB311 passive administration (ranging from a high of 0.35 to a low of 0.05 across experiments)^20^.

There was remarkable consistency between experiments, limited inter-assay variability, with high agreement in flux measurements between experiments among the pooled treatment groups, with an intraclass correlation ranging from 0 to 31.8% (Table 2, Supplementary Fig. 1). Evaluation of vaccine-induced infection reduction (a normalized flux calculated by subtracting the experimental flux from experiment-matched unvaccinated infected controls) found similar variation. Using the adjusted log10 flux may further decrease between-experiment variation compared to the unadjusted flux (Supplementary Table 1). The intraclass correlation for models using the adjusted log10 flux ranged from 0–14.9% (Supplementary Table 1). These results are consistent with inter-assay variability observed for animals receiving AB311 treatment^20^. These results indicate the reduction in liver infection assay can be conducted such that reliable results are obtained between multiple vaccine experiments.

### Relationship between immunization dose and reduction in liver infection

**Fig. 1:**
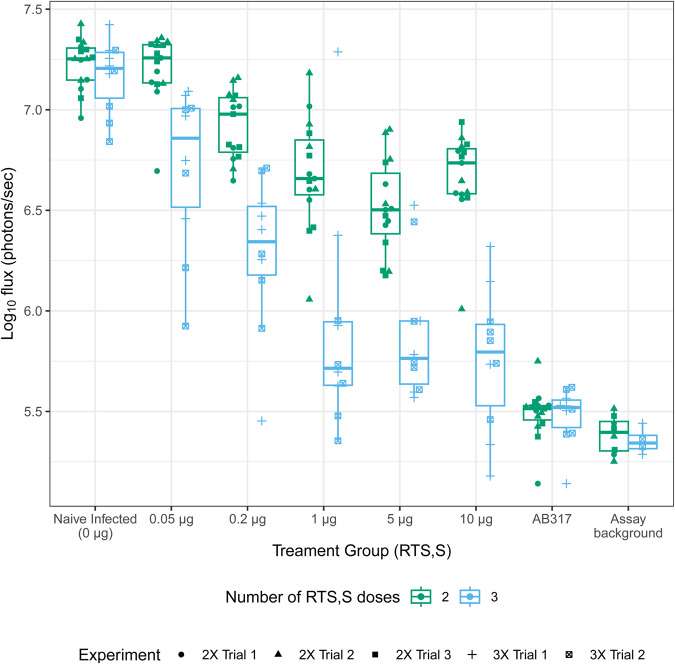
Liver infection measured as Log10 flux among RTS,S dose response (0.05, 0.2, 1, 5, or 10 μg RTS,S) and naïve, infected controls (0 μg RTS,S injected with PBS), by number of doses. Green box plots represent experiments with 2x vaccinations and blue box plots represent experiments with 3x vaccinations. Points are individual animals with shapes used to indicate experiment ID. The mid-line of the boxes denotes the median and the ends of the box denote the 25^th^ and 75^th^ percentiles. The whiskers denote the most extreme data points that were no more than 1.5 times the interquartile range (i.e., height of the box).

The effect of RTS,S/AS01 immunization was measured by assessing flux reduction in immunization cohorts compared to the naïve infected control groups (negative control). Flux reduction in individual animals ranged from none (flux equivalent to naïve infected controls) to strong neutralization (flux reduced to levels close to assay background) (Fig. 1). Increased dose levels of RTS,S using both 2x and 3x administration schedules results in a dose-dependent reduction in liver infection as measured by reduction in flux (Fig. 1, Table 3). All flux reductions were significant except for the group receiving 0.05 μg dose with 2x administration (Holm’s stepwise adjusted *p* < 0.05, Table 3). With a 2x administration schedule, there is a clear limit to this activity achieved at the 5 μg dose level and no improved activity at 10 μg dose level. With the 3x administration schedule, a higher maximum reduction in liver infection was observed that begins to plateau above the 1 μg dose level, achieving a mean reduction of 1.40 log_10_ flux for the 10 μg dose group. For each dose level, a 3x dose schedule significantly reduced liver burden compared to 2x dosing (Holm’s stepwise adjusted *p* < 0.001 for all five doses). Interestingly, compared to the 3x administration at the 10 μg dose level, the 1.72 log_10_ flux reduction elicited by a 300 μg administration of AB317 was significantly lower (Holm’s stepwise adjusted *p*-value = 0.018) (Supplementary Table 2), suggesting that this assay has sufficient range to observe improved reduction in liver infection over RTS,S/AS01.

**Table 3:** Summary statistics for log_10_ flux, and reduction in log_10_ flux among RTS,S/AS01 treatment groups and AB317 group relative to experiment-matched positive controls, for pooled experiments by number of doses and dose level

Number of administrations	Treatment group	Log_10_ flux			Reduction in log_10_flux		
		Median [IQR]	Minimum	Maximum	Mean (95% CI)	*p*-value (unadjusted)	*p*-value (adjusted)
	AB317	5.51 [5.44; 5.53]	5.14	5.75	-1.72 (-1.78, -1.65)		
**2**	0.05 µg RTS,S	7.26 [7.13; 7.32]	6.70	7.36	-0.02 (-0.10, 0.06)	0.603	0.603
	0.2 µg RTS,S	6.98 [6.79; 7.06]	6.65	7.16	-0.31 (-0.39, -0.22)	<0.001	<0.001
	1 µg RTS,S	6.66 [6.58; 6.85]	6.06	7.18	-0.55 (-0.71, -0.39)	<0.001	<0.001
	5 µg RTS,S	6.50 [6.38; 6.68]	6.18	6.90	-0.72 (-0.84, -0.59)	<0.001	<0.001
	10 µg RTS,S	6.74 [6.58; 6.81]	6.01	6.94	-0.56 (-0.69, -0.43)	<0.001	<0.001
**3**	0.05 µg RTS,S	6.86 [6.52; 7.01]	5.92	7.09	-0.45 (-0.71, -0.18)	0.004	0.008
	0.2 µg RTS,S	6.34 [6.18; 6.52]	5.45	6.71	-0.88 (-1.18, -0.58)	<0.001	<0.001
	1 µg RTS,S	5.71 [5.63; 5.95]	5.35	7.29	-1.26 (-1.62, -0.89)	<0.001	<0.001
	5 µg RTS,S	5.76 [5.64; 5.95]	5.57	6.53	-1.28 (-1.53, -1.02)	<0.001	<0.001
	10 µg RTS,S	5.80 [5.53; 5.93]	5.18	6.32	-1.40 (-1.68, -1.13)	<0.001	<0.001

Statistical tests assessing RTS,S/AS01 vaccine effect were performed by comparing each vaccine dose group to the naïve, infected controls by testing whether log_10_ flux reduction was different from zero using linear regression models. All tests were two-sided (alph = 0.05), unadjusted and adjusted (Holm correction) *p*-values are displayed. See Table 2 for sample sizes.

### Induction of CSP reactive antibodies in sera by RTS,S/AS01

**Fig. 2:**
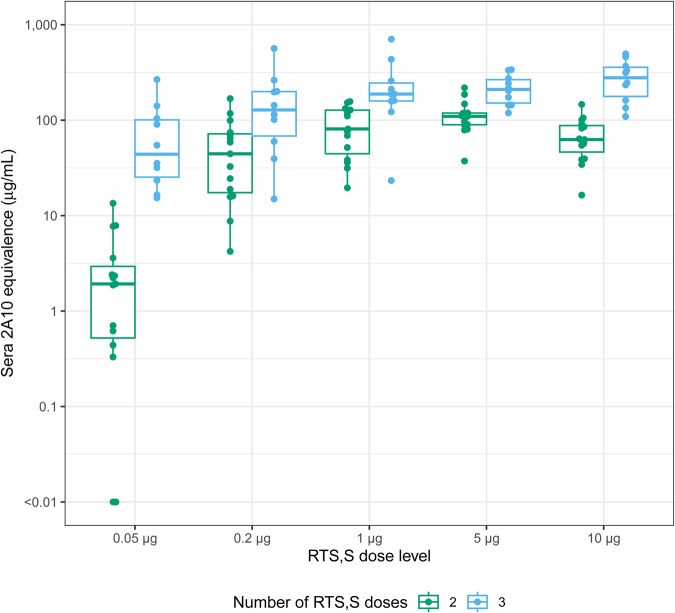
Distribution of RTS,S-induced sera antibody concentrations (2A10 equivalence, μg/mL) by total doses administered and dose levels. Points indicate measurements from individual animals. The mid-line of the boxes denotes the median and the ends of the box denote the 25^th^ and 75^th^ percentiles. The whiskers denote the most extreme data points that were no more than 1.5 times the interquartile range (i.e., height of the box).

To understand how reduction in liver infection related to the levels of anti-CSP antibodies elicited by RTS,S/AS01 immunization, we measured antibody levels in sera prior to challenge. We developed an assay, called the 2A10 equivalence assay, which relates enzyme-linked immunosorbent assay (ELISA) measurements of CSP reactive antibody in sera to ELISA measurements using a standard CSP reactive mouse mAb 2A10^35,36^. This results in the ability to report vaccine induced antibody in mass concentration units (μg/mL) and is a means of ranking responses across experiments. Blood was sampled via retro-orbital bleed 2 days prior to challenge, processed to serum, and subject to ELISA testing. As expected, levels of CS-specific antibody increased with dose and number of RTS,S/AS01 administrations (Fig. 2). Variability in sera antibody concentrations in mice receiving RTS,S/AS01 showed remarkable consistency between experiments when comparing the mean log_10_ titer for each group (Supplementary Table 3).

The intraclass correlation was 0 for all treatment groups, indicating the assay variability was entirely attributable to intra-assay rather than inter-assay variance, except for the pooled 0.2 μg two-dose RTS,S groups (intraclass correlation = 0.533) where there was only moderate agreement between experiments (Supplementary Table 3). Interestingly, there was notable intra-experiment variation for each dose level and administration group. Intra-assay variability showed SDs ranging from 0.157–0.651 (1.44–4.48 fold) (Supplementary Table 3). Encouragingly, this variation in levels of serum antibody produced by RTS,S/AS01 vaccination in these assays does not seem to be due to experimental variation as the intraclass correlation values were very low.

### Relationship between serum anti-CSP antibody concentration and reduction in liver infection

**Fig. 3:**
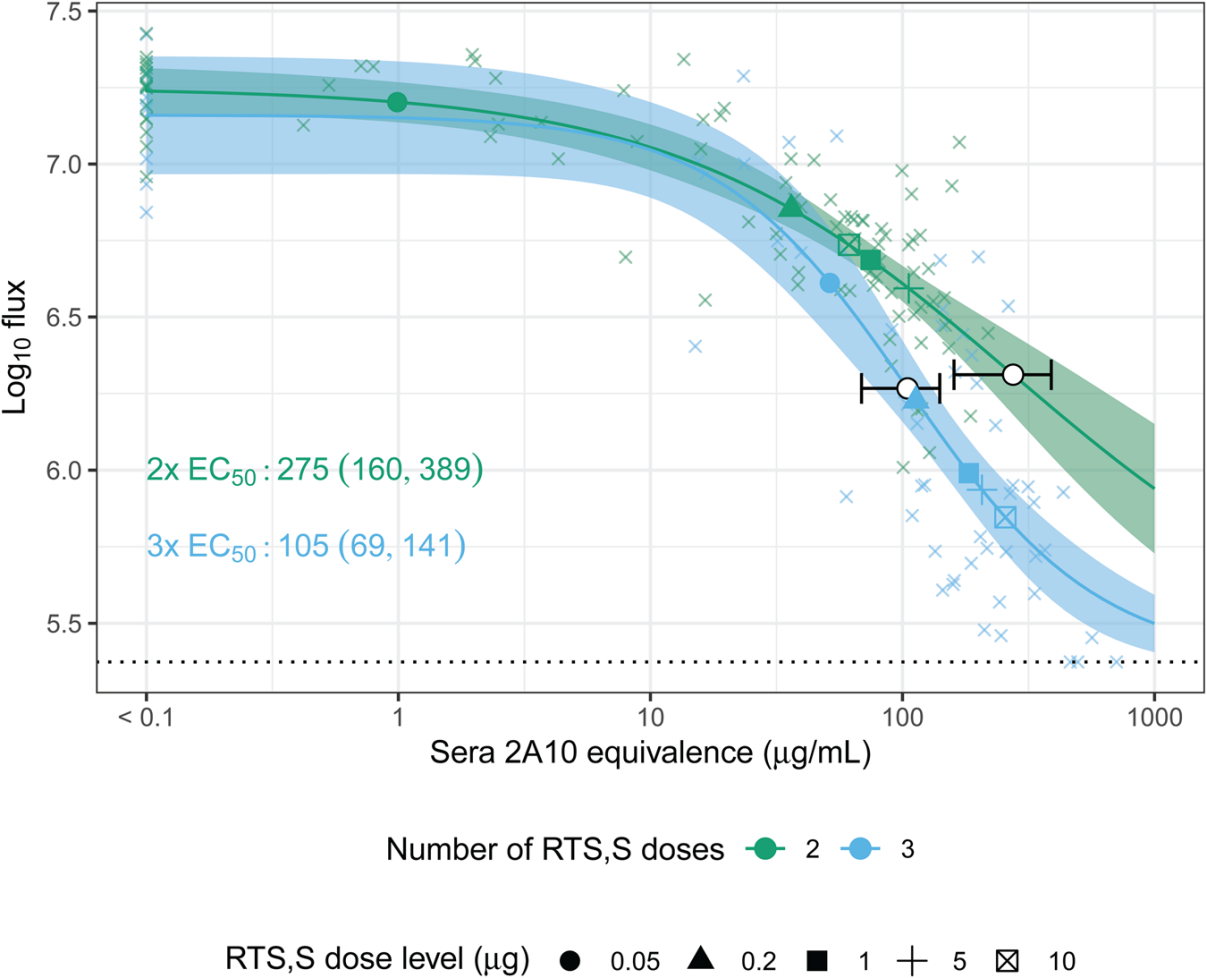
Predicted liver infection measured as log_10_ flux and 95% CI (shaded area) from the 4PL model by total administrations (2 and 3, color), overlaid on observed log_10_ flux values (x’s). Each EC_50_ and its 95% CI are shown with a white point and horizontal line on the 4PL curve. The large points on the curve depict the mean sera 2A10 equivalence (μg/mL) by dose-level (shape) with corresponding predicted flux. The EC_50_ values for the 2x and 3x dose groups were significantly different (*p* < 0.001, two-sided *t*-test). There was a slight vertical difference in EC_50_ log_10_ flux values by dose groups due to differences in experiment-matched infected controls that determine the upper asymptote. Log_10_ flux values below the mean assay background measurements were truncated at 5.37 (dotted line).

We sought to understand the relationship between achieved antibody concentration and inhibition in liver infection across the five experiments (Fig. 3). Sera antibody concentrations measured 2 days prior to challenge showed a non-linear dose response relationship with log_10_ flux. We used a 4 parameter logistic (4PL) model to fit the dose-response relationship for 2x and 3x dose groups and estimate their EC_50_: the effective concentration of sera CSP reactive antibodies required to reach a 50% reduction in log_10_ flux relative to the positive and negative controls. The EC_50_ values for the 2x and 3x dose groups were 275 μg/mL (95% CI: 160, 389) and 105 μg/mL (95% CI: 68.8, 141), respectively; suggesting that the 3x dosing induced antibodies are more potent (*p* < 0.001, two-sided *t*-test). This difference potentially indicates a qualitative difference in antibodies between two and three administration experiments that is not captured by the measured sera concentration as measured by 2A10 equivalence values alone.

### RTS,S/AS01 as a benchmark for future vaccine testing using liver infection comparison studies

**Fig. 4:**
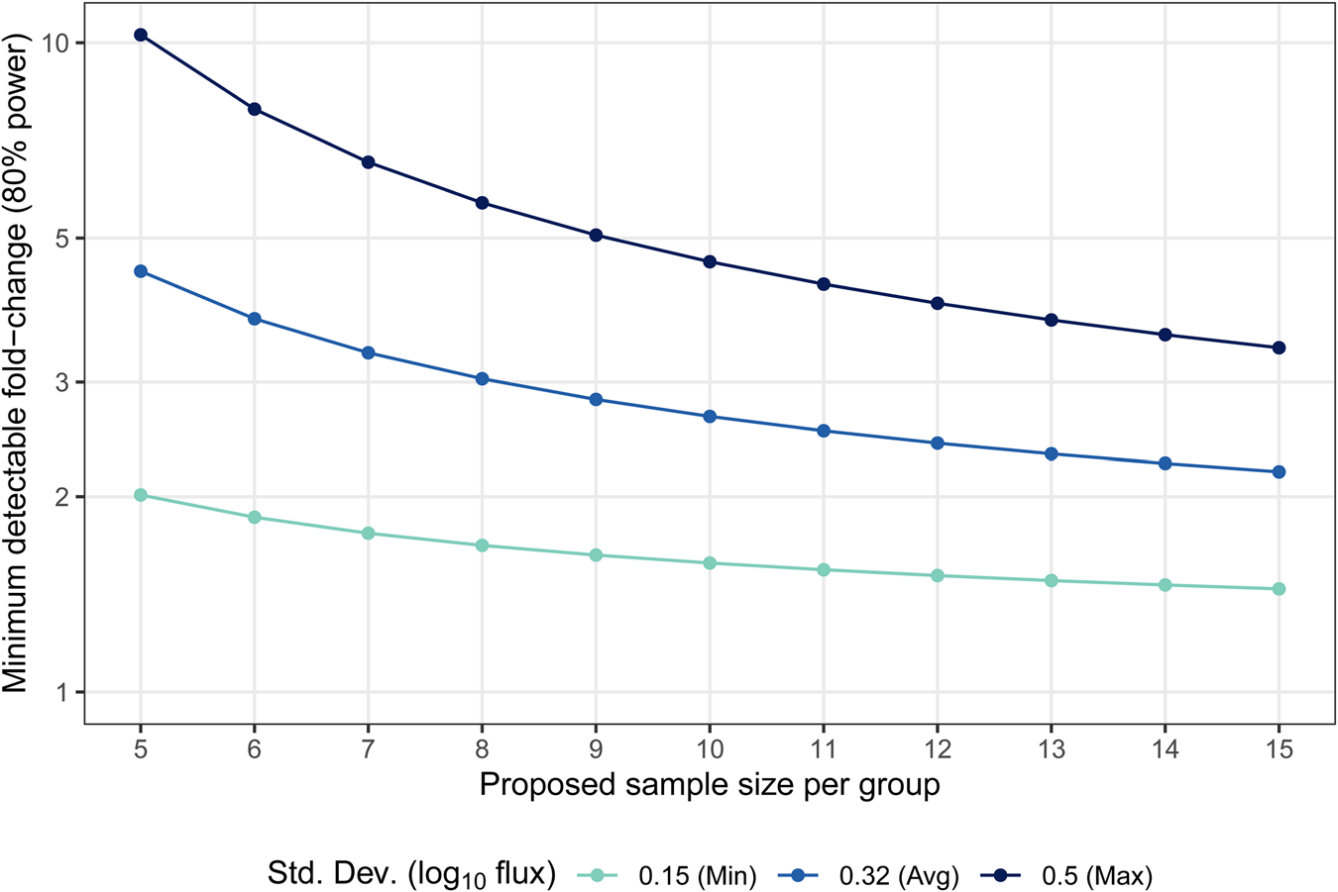
Minimum detectable flux fold-changes with 80% power, using a *t*-test to compare log_10_ flux between RTS,S/AS01 treatment groups, with 5 to 15 mice. Colors denote different input SDs for log_10_ flux.

One of the goals of these models is to enable the testing of new CS-based vaccine candidates to determine if improved potency can be detected. Therefore, calculations were performed to determine the minimum detectable fold-changes in liver infection (log_10_ flux) between groups with 80% power using sample sizes between 5 and 15 mice per group (Fig. 4). Calculations were performed using the minimum, average, and maximum SDs to provide boundaries for the best-, average-, and worst- case variability scenarios. All comparisons were sufficiently powered to detect a >1 log10-flux difference (a 10 fold change) under any setting. Using the average experimental variability, experiments are powered to detect a 5 fold change using at least five mice per group and a 3 fold change using at least eight mice per group, whereas detection of a 2 fold change is only possible with *N* = 5 under the best-case variation scenario (Fig. 4). These calculations indicate the importance of measuring and limiting assay variability when conducting comparison studies.

### Sterile protection from mosquito bite challenge: assay consistency

An endpoint that more closely mimics natural exposure and controlled human malaria infection (CHMI) application is the ability of a vaccine to protect from blood-stage parasitemia following an exposure via mosquito bite challenge. The analysis of this model relied on data from four experiments each with 35 mice randomized to 0.05, 0.2, 1, 5, or 10 μg RTS,S (seven mice per dose level) (Table 4). As in the liver infection experiments, all mice received a constant amount of adjuvant (10 fold diluted original AS01_E_ adjuvant dose, representing 2.5 µg of the TLR4 ligand 3-*O*-desacyl-4’-monophosphoryl lipid A (MPL), and 2.5 µg of the QS-21 saponin). Seven positive control mice received 300 μg AB317 and seven mice received PBS as the naïve infected control. In three experiments, the animals received three vaccinations, and in the remaining experiment they received two vaccinations. All vaccinations were administered IM at 3-week intervals as was done with liver burden experiments. Mice were challenged by five tg*Pb-Pf*CSP infected mosquito bites 2-weeks after the last immunization. Blood was sampled via retro-orbital bleed 2 days prior to challenge, and sera CSP antibody concentrations were measured by ELISA. On days four through ten post challenge, blood smears from the tip of the mouse’s tail were collected to determine the presence of blood-stage parasites.

**Table 4:** Experimental design and protection results of four mosquito bite challenge experiments

Experiment ID	Total administrations	AB317	Naïve (no vaccine)	RTS,S dose levels				
				0.05 μg	0.2 μg	1 μg	5 μg	10 μg
RTS,S 2x	2	5/7 71.4%	0/7 0%	0/7 0%	0/7 0%	0/7 0%	0/7 0%	1/7 14.3%
RTS,S 3x-1	3	5/7 71.4%	1/7 14.3%	3/7 42.9%	3/7 42.9%	3/7 42.9%	4/7 57.1%	3/7 42.9%
RTS,S 3x-2	3	4/7 57.1%	0/7 0%	0/7 0%	0/7 0%	1/7 14.3%	5/7 71.4%	1/7 14.3%
RTS,S 3x-3	3	6/7 85.7%	0/7 0%	1/7 14.3%	2/7 28.6%	3/7 42.9%	4/7 57.1%	6/7 85.7%

Results are shown as the fraction and percentage of protected mice over the number challenged. Experimental ID represents the immunogen (RTS,S), the number of administrations (eg 2x = two administrations, and the individual experiment number (eg -1)). For the AB317 groups, only one administration was given 16 h prior to challenge.

In general, increasing the RTS,S dosage and the number of immunizations conferred increased sterile protection (Table 4). Mice receiving two doses, regardless of the dose level, became infected with the exception of one mouse out of seven immunized with 10 μg RTS,S (14.3% protected). Sterile protection was observed in mice receiving three doses, with a higher proportion of mice protected in the higher dose groups (1, 5, and 10 μg). Of note, the highest protection overall was seen in the 5 μg group (61.9% protection average across the three 3x experiments) which was higher, but not significantly (*p* = 0.526, Barnard’s exact test), than the 10 μg group (47.6%). The pattern of protection varied among the three experiments (Supplementary Fig. 2), which highlights the challenges of the protection from mosquito bite challenge model. Consistency of results in this model is harder to maintain due at least in part to inherent variability in the number of sporozoites introduced by each mosquito bite. In one experiment denoted 3x-3, a dose response relationship of increasing protection with increasing vaccine dosage was observed, however in the other two experiments, the 5 μg dose conferred the greatest protection (Supplementary Fig. 2).

### Levels of anti-RTS,S antibody in sera in mosquito bite challenge experiments

Using the 2A10 equivalence assay, we estimated the levels of CSP-reactive antibodies circulating in the mouse at the time of mosquito bite challenge followed similar patterns as those observed for the reduction in liver infection. As previously observed, in all four experiments analyzed, both for two and three administrations of vaccine, antibody concentration rose with increasing dose level up to 5 μg RTS,S where it reached a plateau (Supplementary Fig. 3).

### CSP-reactive antibodies as a predictor of protection from mosquito bite challenge

**Fig. 5:**
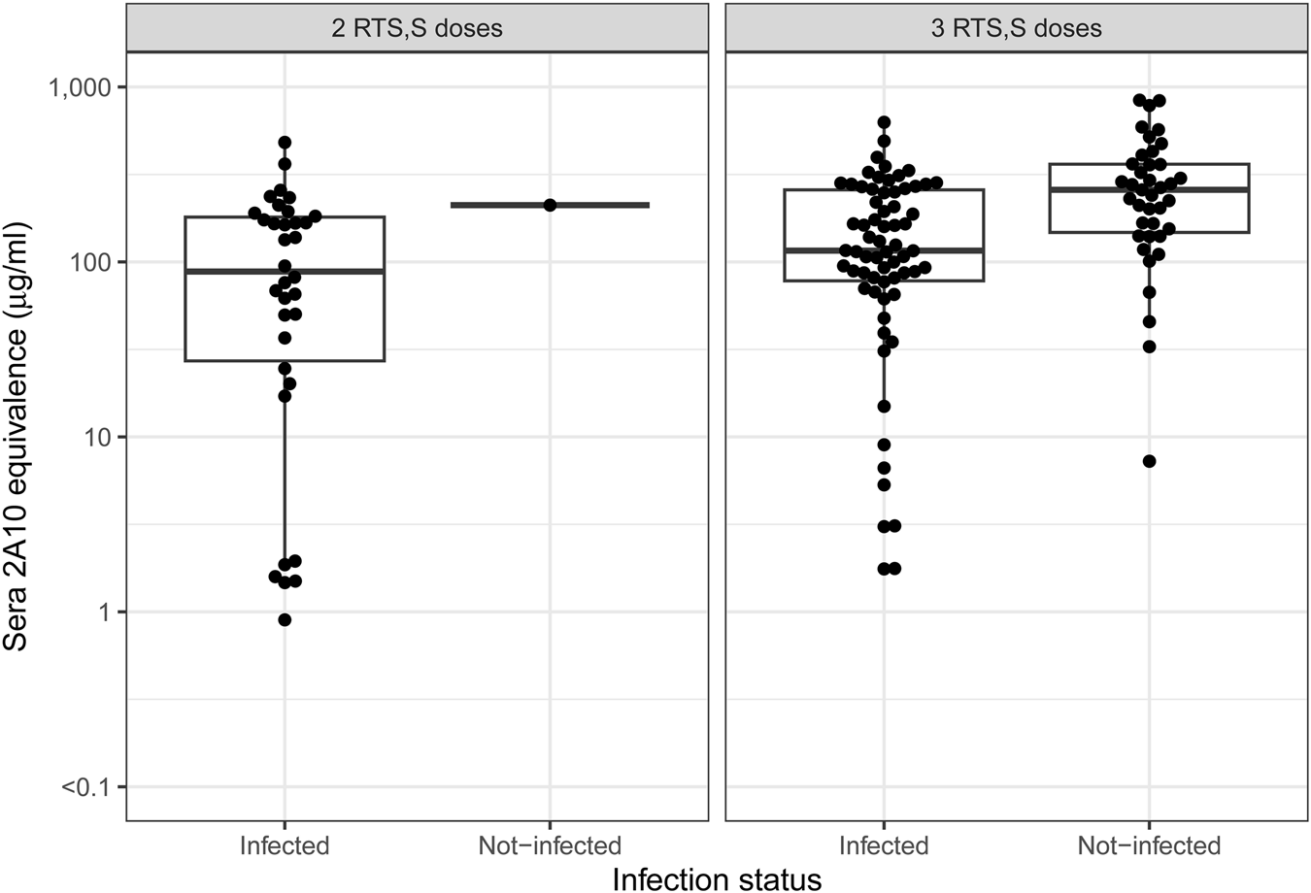
Distributions of antibody concentration measured as sera 2A10 equivalence (μg/mL), measured 2 days prior to challenge, by infection status, by two and three administration experiments. The mid-line of the boxes denotes the median and the ends of the box denote the 25^th^ and 75^th^ percentiles. The whiskers denote the most extreme data points that were no more than 1.5 times the interquartile range (i.e., height of the box).

Antibodies to CSP have been implicated as a key component of a protective immune response induced by RTS,S/AS01 in CHMI studies^6,9,37–41^, and consequently, we sought to understand to what extent sera antibody levels can predict protection from mosquito bite challenge. In the three administration experiments, sera antibody levels were on average higher in the protected mice compared to the infected mice (Fig. 5). As with the liver infection experiments, there appears to be a qualitative difference between the antibodies generated by two and three administrations; here, despite some comparability in ranges of sera antibody concentrations, experiments with two administrations elicited no protection.

**Fig. 6:**
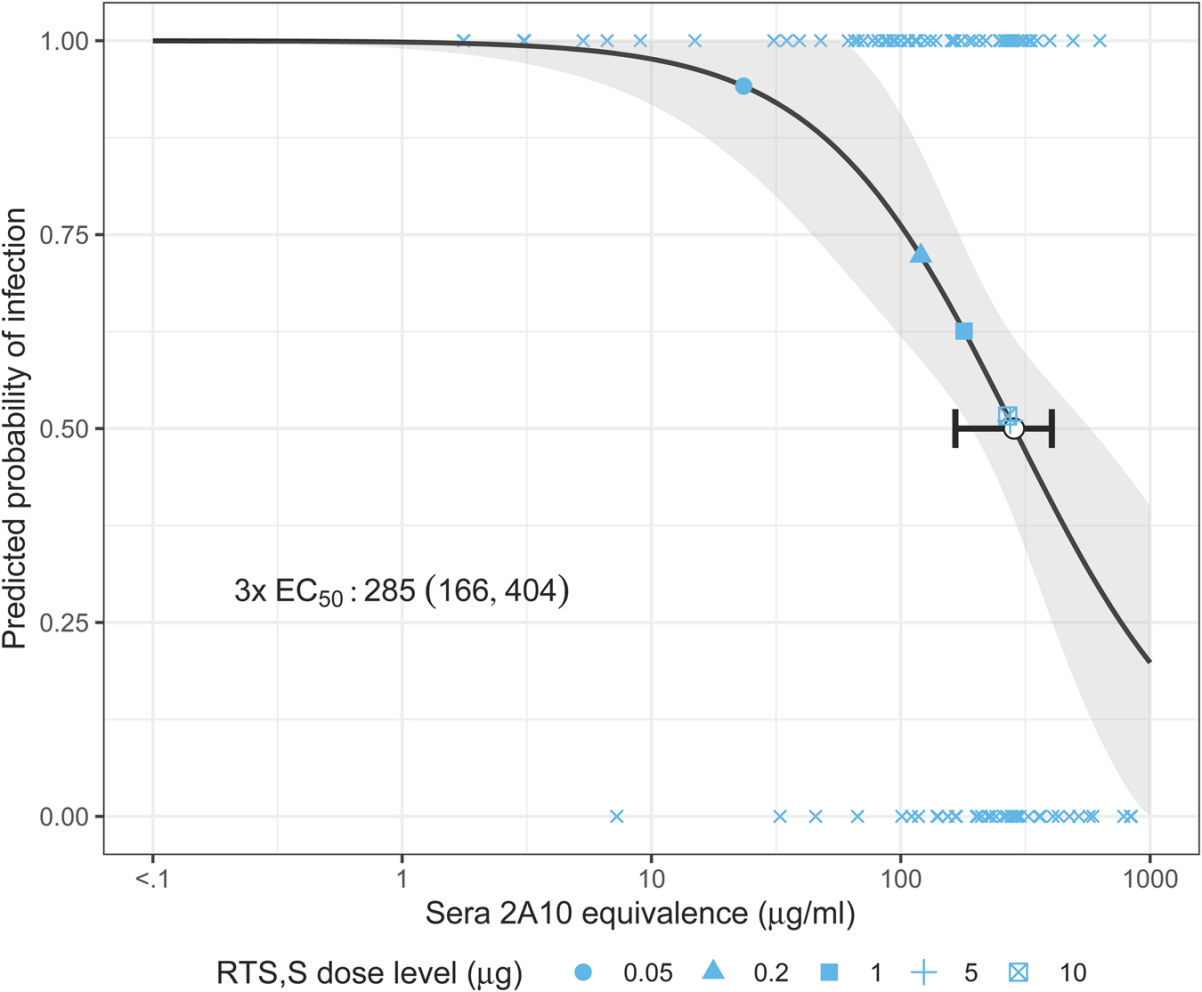
Predicted probability of infection and 95% CI (shaded area) for varying range of sera antibody concentration measured as 2A10 equivalence (μg/mL) based on the logistic model, overlaid on observed infection status (x’s) for the three administration experiments (black curve with gray shaded area). The EC_50_ and its 95% CI are shown with a white point and horizontal line on the 2PL curve. The blue points on the curve depict the mean sera 2A10 equivalence (μg/mL) by dose-level (shape) with corresponding predicted infection probability. The symbols for the 5 and 10 μg dosages are overlapping on the curve.

To determine whether there was a dose response over serum antibody levels for the experiments using three administrations, infection probabilities were assessed within each sera antibody level quintile. As expected, there was an inverse relationship between proportion of infected mice and quintiles of serum antibody levels (Supplementary Fig. 4). We next used a logistic model to fit the dose-response relationship between sera CSP reactive antibodies and infection probability for the three administration experiments (Fig. 6). The EC_50_ estimate was 285 μg/mL (95% CI: 166, 404) based on the three administration experiments measured as 2A10 Equivalence units. This indicates to reduce infection probability to 50%, antibody concentration in the 250–300 μg/mL range is necessary. This is similar to the mean concentrations induced by the 5 and 10 μg groups with 3x dosing (Supplementary Fig. 4).

### RTS,S/AS01 as a benchmark for future comparison studies using mosquito bite challenge

**Fig. 7:**
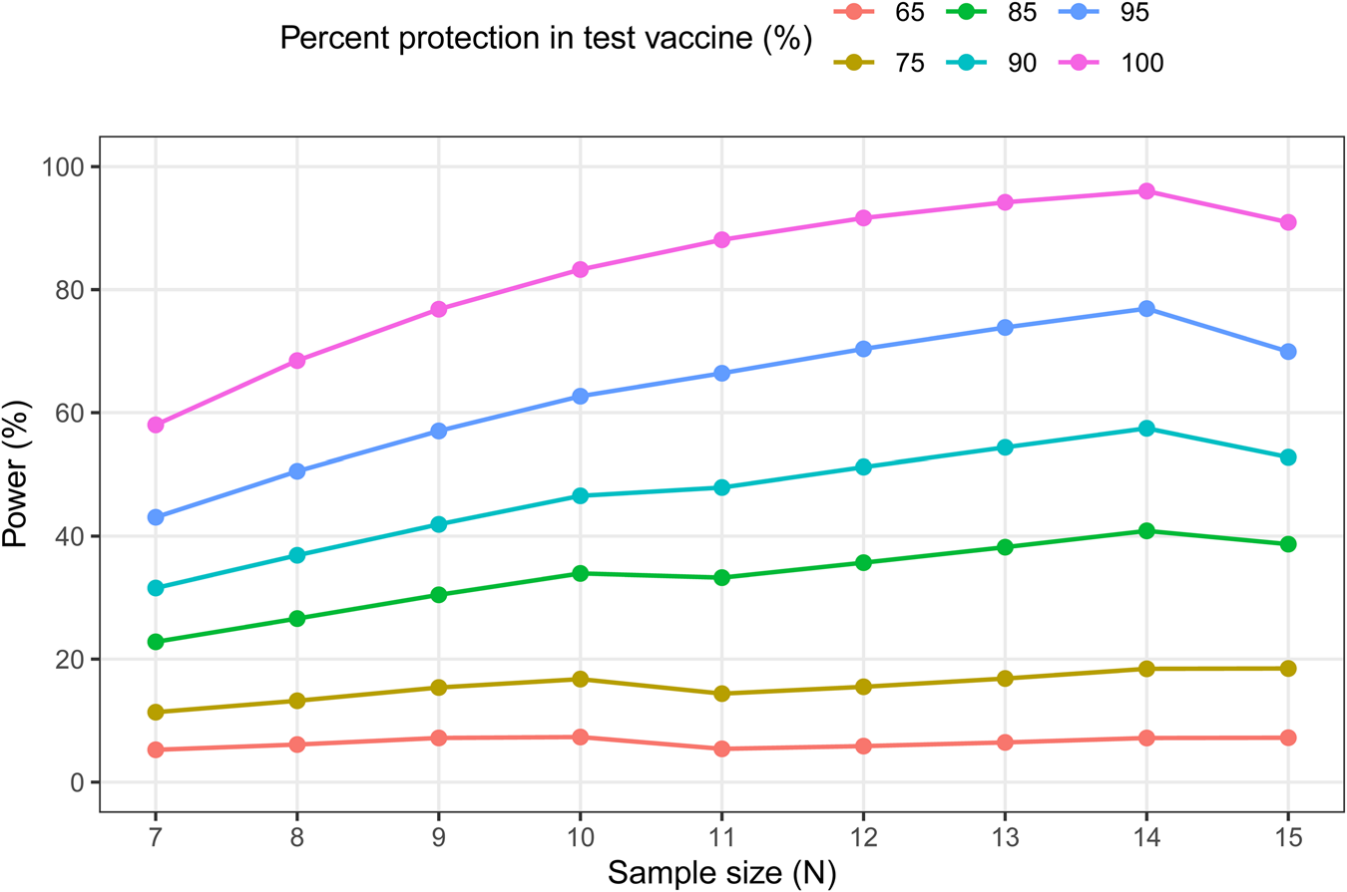
Statistical power from Barnard’s test comparing superiority of test vaccine with protection levels ranging from 65% to 100% to three administrations of 5 μg RTS,S protection of 60% by sample size (*N* = 7–15). For exact tests comparing binary outcomes, power may decrease at increments of sample size increases.

We next explored how the protection from the mosquito bite challenge model might be used to compare novel vaccine candidates relative to the RTS,S/AS01 benchmark. We considered future scenarios in which a hypothetical test vaccine is capable of conferring 90%, 95%, and 100% protection and compared this to the 60% protective three administrations of the 5 μg RTS,S treatment group using the Barnard two-sided test (chosen for its adaptability to one-sided non-inferiority testing) (Fig. 7). To simplify this modeling assessment, we assumed to have parasitemia detected on day six post-challenge; we recognize this is an oversimplification as infections were detected as early as day four and as late as day seven and some information might be obtained by considering delay to parasitemia. In the case of strict superiority, a sample size of ten mice per group will achieve at least 80% power for detecting superiority of a test vaccine conferring 100% protection compared to three administrations of 5 μg dose RTS,S with 60% protection. For a test vaccine conferring protection levels of 90% or below, the power to detect superiority is <60% even with the highest sample sizes tested (*n* = 15).

### The reduction in liver infection model can differentiate between protected and non-protected human samples

Having established the parameters for use of the challenge model in benchmarking potency to RTS,S/AS01, we evaluated the model’s ability to differentiate between protected and non-protected human plasma samples. If these murine models are to be used for down-selection of CSP based vaccine candidates prior to clinical testing, they should be able to differentiate between samples from protected and non-protected participants from CHMI trials. Plasma was obtained from individual subjects immunized with RTS,S/AS01 (50 μg RTS,S, 50 μg AS01) on a 0, 1, and 7 month regimen, with a fractional (1/5) final month 7 dose. Volunteers were then challenged by infected mosquito bite 3 months after the final immunization. The two groups that provided samples for this preclinical study are from the AduFx and 2PedFx groups, which received the adult dose or a double pediatric dose, thus the same amount of antigen and adjuvant, on a 0, 1, 7 month schedule. These groups displayed 55% and 75% vaccine efficacy, respectively, in the CHMI challenge^42^.

**Fig. 8:**
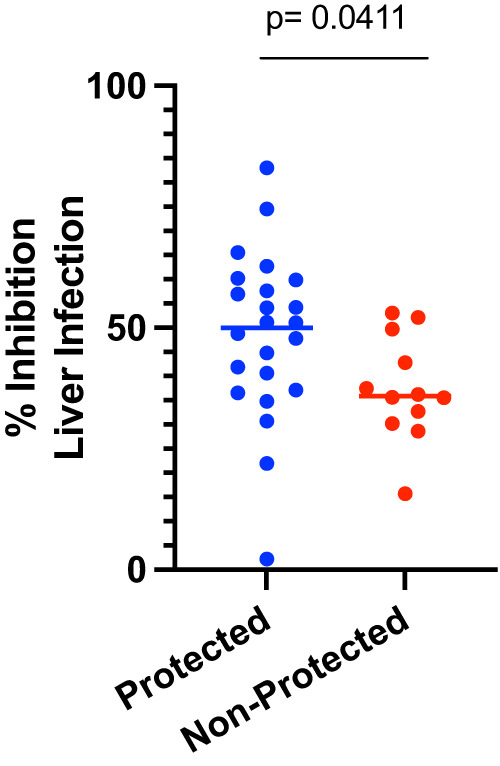
Percent inhibition of liver infection conferred in mice receiving via passive transfer 500 μL of individual day of challenge human plasma samples from protected (*n* = 24) and non-protected (*n* = 12) subjects in the AduFx and 2PedFx cohorts receiving the adult dose or a double pediatric dose, thus the same amount of antigen and adjuvant, on a 0, 1, 7 month schedule of the MAL092 CHMI study. Plasma from protected subjects conferred significantly greater inhibition of liver infection than plasma from non-protected subjects. (Mann-Whitney U test).

Individual 500 µL plasma aliquots collected on the day of sporozoite challenge were pooled by protection status and IV injected into mice at two doses, 300 μL and 500 μL, followed by IV sporozoite challenge 16 h later using protocols previously reported for the administration of mAbs^20^. Monoclonal AB311^32^ (300 μg) and mice receiving only PBS prior to challenge (naïve) were included as positive and negative controls, respectively. Levels of liver infection as assessed by flux measurements were measured and a statistically significant was observed between liver infection in mice who received pooled plasma from protected subjects and naïve mice. In contrast, there was no significant difference in liver infection between both mice receiving pooled plasma from non-protected subjects and mice receiving naïve human plasma compared to naïve mice (Supplementary Fig. 5). Encouraged by this result, we then passively transferred 500 μL plasma aliquots IV, this time from individual subjects, into mice, challenged IV, and observed a statistically significant difference between mice who received plasma from protected subjects compared to non-protected subjects. (*p* *=* 0.0411, Mann-Whitney U test, Fig. 8). In the group that received the plasma from non-protected subjects we observe some inhibition of liver infection as expected as they have antibodies against CSP. This result indicates these murine models of *P. falciparum* infection can emulate differences seen in the clinic, and these models are relevant to CSP-based vaccine candidate evaluations.

## Discussion

The results reported here provide a foundation for using a mouse model of *Plasmodium* infection to compare vaccine candidates against a clinically relevant standard, RTS,S/AS01. Similar to previous reports for testing mAbs^20^, inter- and intra-assay variability can be minimized and maintained within acceptable bounds for both the reduction of liver infection following IV sporozoite injection and sterile protection following mosquito bite challenge. Rigorous maintenance of experimental protocols for preparation of sporozoites and in the conduct of the assay is likely key to this outcome. As all the results reported here were conducted in one laboratory, inter-laboratory variability of these assays will be important to understand in the future. As reported previously for testing mAbs, assessment of assay variability is critical and should be monitored in future experiments as this will determine the required group size of experiments and have bearing on the reliability of the data^20^. When comparison studies using RTS,S/AS01 are conducted, at least two different dose levels of RTS,S should be included to better fit the 4PL curve used to model the relationship between sera antibody concentration and reduction in liver infection in the studies reported here.

There is particular consistency in inter-assay results using the liver infection assay across various dose levels and two schedules of RTS,S/AS01. This result occurs despite the fact there is evident variability in the induced level of antibodies in individual mice at every dose level and schedule. The results on intra-assay comparability confirm that a feasible group size can be used to detect differences as indicated in Fig. 4, Table 2, and Supplemental Table 1. Given this consistency and experimental feasibility, we propose the protection from liver infection assay be the primary testing tool for detection of activity of a novel vaccine candidate and comparison to the RTS,S/AS01 benchmark. The protection from mosquito bite challenge assay also provides important information and should be employed, but our results indicate that novel vaccine candidates will need to achieve very high protection in order for experiments to be designed with adequate power to determine a difference from RTS,S/AS01 given with three administrations.

The inter- and intra-assay variability as well as the concentrations of antibody needed for protection reported here may not extend to other similar mouse models of infection since the models differ in potentially important ways. For example, differences in the strain of mouse or the engineering of the transgenic sporozoite could result in differences in the efficiency of infection. Similarly, reports on the use of these models show differences in the experimental conditions for challenge, such as the dose of infectious sporozoites or the route of administration, possibly leading to different levels or variability of infection. To our knowledge, the current and previous studies^20^ are the first reported attempts at assay qualification. Since our results indicate assay variability can have a large impact on the ability to reliably detect differences in the effectiveness of vaccines and antibodies, we recommend these parameters should be determined when alternate experimental configurations of these murine models are used.

Other mouse models have been developed to study infection and test vaccines and antibodies. One approach uses intact *P. falciparum* sporozoites infecting immunocompromised FRG hu Hep mice^43,44^. Since this model has been used to test mAbs, it could potentially be used to test vaccine-elicited polyclonal sera but is likely not useful for active vaccination studies because of their deficient immune responses to vaccination. Furthermore, studies of human antibody response are possible using vaccination in mice that contain the human antibody repertoire^45^. These mice have been used to generate human mAbs^46^, study affinity maturation^47^, and study in vitro functional activity^48^, but not to date as infection models.

Two recent studies report immunization using RTS,S/AS01 in a mouse model^31,49^. In the first study, RTS,S/AS01 was compared with the similar vaccine R21/Matrix-M. Comparable levels of sterile protection conferred by the two vaccines was observed; these levels were similar to that measured in our study following two administrations of RTS,S/AS01^49^. We hope that a side-by-side comparison of RTS,S/AS01 with other vaccines, including R21/Matrix-M, may be possible in the near future. In the second and more recent study^31^, RTS,S/AS01 was used to compare various ratios of immunogen to the same adjuvant dose as reported in the present study. Results are similar between the two reports in that a plateau of antibody levels at similar concentration of immunogen is observed when three administrations were given. Sterile protection was assessed but using a different challenge protocol which used intravenous challenge with sporozoites rather than mosquito bites. Interestingly similar EC_50_ values were reported in liver infection experiments in the second study and our study, but the EC_50_ calculated from the protection from parasitemia experiments was higher in our study, possibly due to the different route of administration. As reported here, protection did titrate with antigen at constant adjuvant dose. The second study also explores the effect of vaccine titration at a fixed ratio of antigen to adjuvant; such analysis is not reported here. The present study is unique in that RTS,S/AS01 induced responses are characterized in the more quantitative liver infection model. Differences between these two studies and the current study include use of a different construct to create the *P. falciparum* expressing *P. berghei* sporozoites, a higher level of adjuvant, and a different mouse strain, as well as conducting the challenge using IV administration of sporozoites rather than mosquito bite. These studies suggest that each configuration of the mouse infection model requires care in standardization.

This and similar mouse challenge models may not be suitable for screening all malaria vaccine candidates currently under development. This model as it exists at present is only relevant to CSP-based vaccine candidates, although it may be possible to generate transgenic parasites to support assessment of vaccine candidates based on other malaria antigens. A vaccine based on radiation-attenuated sporozoites is showing efficacy in clinical testing. The activity of this vaccine candidate is attributed to induction of both antibodies and cellular immune responses^50,51^ and consequently may need very different models for those reported here for preclinical testing.

Future vaccine candidates, including those genetically delivered, may vary in their use of adjuvant and the mode of antigen delivery. However, for vaccine candidates targeting CSP, the level of protection against infection will likely continue to be generally related to the serum concentration of anti-CSP antibodies. This has also been observed in humans^6,9,37–41^ where antibody concentration falls over time likely leading to reduced protection^9^. Therefore, a reasonable goal of vaccine design should be induction of antibodies having higher quality, that is, elicitation of anti-infection activity at lower concentrations and, as a result, prolonged protection. Our calculations of EC_50_ levels for RTS,S/AS01 induced antibody provide guidance for the ability to detect differences in antibody quality. For the reduction in liver infection assay, our power analysis calculations indicate that three-fold differences of log_10_ flux of a test vaccine compared to RTS,S/AS01 should be detectable using reasonable group sizes.

**Fig. 9:**
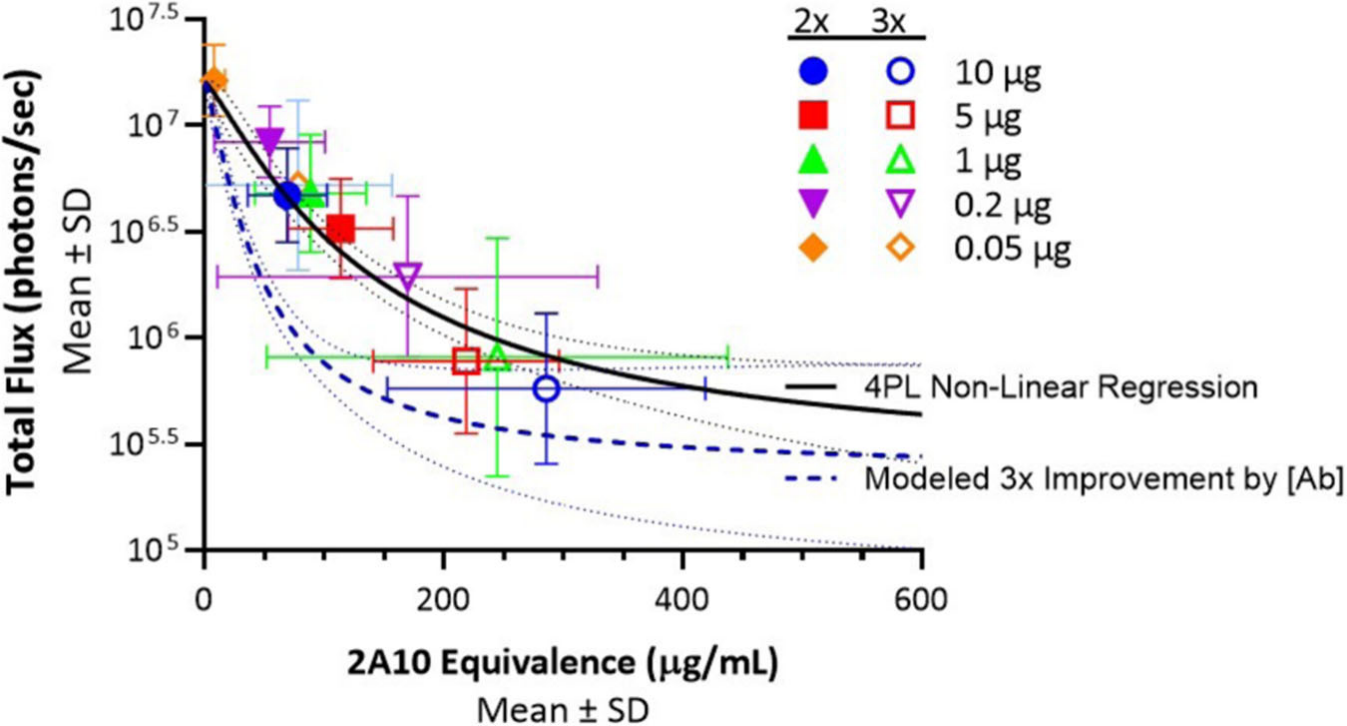
Total flux (photons/second) plotted against sera 2A10 equivalence (μg/mL) and fit to a 4PL regression curve. The 4PL fit is shown in the solid black line, with 95% CIs depicted by the dotted lines. The modeled three-fold improvement is shown in the dotted blue line, also with 95% CIs.

To help visualize how results from a novel vaccine candidate might be analyzed, we plotted data from all experiments reported here showing levels of reduction in liver burden and levels of induced antibody (Fig. 9). Also plotted on this figure is a curve that presents the performance of a hypothetical next-generation vaccine candidate that induces antibodies that are three-fold more potent; that is, it induces responses that are as functional at one-third the 2A10 equivalence concentration of those elicited by RTS,S/AS01. As shown, the 95% CIs do not overlap for much of the curve, suggesting the potential to demonstrate such a difference using appropriate statistical tests comparing flux or sera antibody concentration between various doses. Experiments using these criteria for success are now in progress for several candidate vaccines. Ideally, such candidate vaccines would be tested with the adjuvant used for clinical development. This single variable approach to testing may help in settings where the adjuvants perform differently in mice and humans.

One limitation of the present study is that we cannot determine whether anti-RTS,S antibodies induced in the mouse are of the same quality as those induced in humans. To achieve this comparison, we would need to have common standards that measure antibody concentration transcending species differences. Unfortunately, at present, the assays rely on “equivalence assays” like the one reported here using the mouse antibody 2A10 as the standard while studies in humans use a human mAb such as AB311 or AB334 as the standard. As such, we cannot directly compare antibody concentration results using these different standards. Development of assays that may resolve this challenge is underway and will allow comparison of the potency of antibodies induced in mice and humans. When these methods are in place, researchers should be able to acquire valuable information on the functional activity of polyclonal sera induced by various candidate vaccines and to compare polyclonal sera to the highly potent mAbs now in human efficacy trials. For now, our finding of increased protection using plasma from protected human subjects in a challenge protection trial of RTS,S/AS01 compared to plasma from non-protected subjects supports that this mouse liver infection model is measuring a meaningful endpoint. The fact there was a low level of reduction in liver infection observed for the non-protected human plasma samples can be expected because there are anti-CSP reactive antibodies in these plasma samples that were able to affect a delay to patency clinically^42^. However, the significantly greater level of reduction in liver burden observed in mice passively vaccinated with plasma from protected human volunteers indicates this preclinical model can measure meaningful clinically relevant functional differences.

In this study, we have focused on functional antibody responses as findings from human serology studies suggest that T-cell responses are not the primary driver of protective responses induced by RTS,S/AS01^39,40^. However, in future work, analysis of T-cell responses in mice immunized with RTS,S/AS01 and other CSP-based vaccines could be performed, enabling further comparison between different vaccines.

The results reported here also add to our understanding of protection by RTS,S/AS01 in humans. These studies indicate that the two-administration schedule is much less effective than the three-administration schedule. This is seen in both the inhibition of liver infection and in the protection from mosquito bite challenge models. For the two-administration schedule, there is a clear plateau in both the level of achieved antibody concentration (Fig. 2) and in the ability to block liver infection (Fig. 1) with almost no protection against mosquito bite challenge. These results are analogous to clinical testing of RTS,S/AS01 in which three administrations were found to be more effective than two^42^. In this study, it is not possible to definitively determine if there is a decrement in antibody quality in the two-administration schedule, as well as the clearly decreased quantity of antibody induced. The structure of the RTS,S immunogen may help explain why. Some researchers have proposed the repeat region of CSP acts in immune evasion^52^ possibly by limiting responses to other regions^26,28^ or diminishing B-cell maturation^37,53,54^, however other investigators have suggested that focusing the immune response via an NPNA-specific vaccine might have advantages^27^. Limitations in peak antibody titer or functional quality as reported here might also be a consequence of the repeated nature of the NPNA immunogen. Determining if this maximum in antibody concentration occurs for antibodies against both the NPNA repeat region as well as other regions, such as the C-terminal region and/or minor repeats, would be interesting. The three-administration schedule also produces a similar if higher maximum level of achieved antibody and functional activity. Our results also suggest the level of functional activity to prevent liver infection achieved by 5 μg or 10 μg dose levels of RTS,S delivered with three administrations reaches a plateau, and this is less than the level seen with a high dose of AB317 and less than the dynamic range of the assay as defined by the uninfected controls. This is seen in all individual experiments and when the data are summed across all three experiments. This finding may identify an important goal for future vaccine designs, and highlights the potential of these models for screening promising new CSP-based vaccine candidates that could have greater functional potency than RTS,S/AS01.

## Methods

### Animals

Mouse studies used 6–8-week-old C57BL/6 female mice (Charles River Labs, Frederick, MD, USA), maintained at the animal facility of the Johns Hopkins Bloomberg School of Public Health. Mouse housing was maintained at 40–60% relative humidity and a temperature of 68–79 F, with at least ten room air changes per hour and a 14/10-h light/dark cycle. No animals or data points were excluded from analyses. Experiments were performed in strict accordance with the recommendations in the Guide for the Care and Use of Laboratory Animals of the National Institutes of Health. The protocol was approved by the Animal Care and Use Committee of Johns Hopkins University (protocol numbers MO18H419 and MO21H417). For in vivo procedures such as bleeding and imaging, mice were under partial anesthesia, (isoflurane), for the mosquito bite challenge, mice were under deep anesthesia (2% avertin). After the final readout, mice were euthanized by CO_2_ exposure (5 min), followed by cervical dislocation, following guidelines at JHU.

#### Antibodies

AB311 and AB317 are human immunoglobulin G1 (IgG1) mAbs isolated from experimental clinical trial of RTS,S/AS01 MAL071^41^ and bind to NPNA repeats^32^. They were expressed by transient transduction 0.5 L TunaCHO cultures followed by protein A purification at Lake Pharma Inc. Belmont, CA.

2A10 is a mouse mAb specific for *P. falciparum* CSP^36^ that recognizes the repeat domain of this antigen^55^.

#### RTS,S/AS01_E_

Pediatric doses of RTS,S/AS01_E_ (Mosquirix^TM^) were kindly provided by GSK, Rixensart, Belgium. AS01_E_ is an Adjuvant System containing MPL, QS-21 and liposome (25 µg MPL and 25 µg QS-21 per 500 µl dose).

#### Naïve plasma

Naïve human plasma was kindly provided by Dr. David Sullivan, Bloomberg School of Public Health, JHU. For experiments, a pool of 5 naïve plasma samples was used.

#### Immune plasma

Individual day of challenge plasma samples from protected (*n* = 24) and non-protected (*n* = 12) subjects in the AduFx and 2PedFx cohorts, receiving the adult dose or a double pediatric dose on a 0, 1, 7 month schedule, of the MAL092 RTS,S/AS01 CHMI study^42^ were kindly provided by GSK.

#### Parasites

Transgenic sporozoites in *P. berghei* expressing *P. falciparum* CSP, green fluorescent protein and luciferase reporter gene, used in all studies, has been previously described^18^. Parasite preparation has been described in detail^18^. Briefly, 5 day old adult *Anopheles stephensi* mosquitoes were allowed to feed on mice carrying 1 to 2% transgenic parasites. Then, 20–22 days post murine blood meal, transgenic sporozoites were collected from salivary glands and used within 60 min for IV infection for liver burden studies. For parasitemia studies, infectious mosquitoes were used directly.

### Reduction in liver infection assay

Challenge studies assessing reduction in liver infection were conducted as previously described for passive transfer of mAbs^18,20^. Mice were immunized with a range of concentrations of RTS,S/AS01 either two or three times at 3-weeks intervals via IM injection into alternating *M. tibialis*. Each mouse was immunized with 50 μL of the designated treatment, which spanned a range of 0.05, 0.2, 1, 5, and 10 µg RTS,S, with adjuvant dose held constant (10 fold diluted original AS01_E_ adjuvant dose, representing 2.5 µg of the TLR4 ligand 3-*O*-desacyl-4’-monophosphoryl lipid A (MPL), and 2.5 µg of the QS-21 saponin) The negative control cohort received injections of 50 μL PBS on the same schedule. The positive control cohort received 300 μg of AB317 in PBS via passive transfer IV in the tail-vein 16 h prior to challenge. Sera samples were taken via retro-orbital bleeding 2 days prior to challenge. ELISAs were performed on all samples. Animals were challenged IV with 2,000 *P. berghei* (*Pb*) transgenic sporozoites that express full length *Pf* CSP and GFP-Luciferase (GFP-Luc) enzyme 2-weeks after the last immunization. Forty-two hours after parasite challenge, parasite load in the liver was measured by bioluminescence in an in vivo imaging system (IVIS Spectrum, Perkin Elmer). Mice were injected intraperitoneally with 100 μL of d-luciferin (30 mg/mL) and immediately anesthetized with isoflurane for 5 min prior to IVIS. Groups of five anesthetized mice were placed in the imager and the radiance measurements recorded by the live imager software, version 4.5.1. The total flux reading for each mouse was recorded individually. Background reading was verified for each study with two naïve mice that received only the d-luciferin substrate. the radiance measurements recorded by the live imager software, version 4.5.1. The total flux reading for each mouse was recorded individually. Background reading was verified for each study with two naïve mice that received only the d-luciferin substrate.

### Protection from mosquito bite challenge assay (parasitemia assay)

Challenge studies assessing protection from parasitemia were conducted as previously described for passive transfer of mAbs^18,20^. *An. stephensi* mosquitoes were fed on mice infected with chimeric *P. berghei* parasites encoding full-length *P. falciparum* CSP. In order to determine the proportion infected, 19–20 days after blood feeding on mice, a few mosquitoes were dissected to determine whether sporozoites were present in salivary glands. The proportion of mosquitoes infected was used to inform the number of mosquitoes exposed to each mouse, i.e., if 80% of the mosquitoes were infected, six would be exposed to each mouse. Two weeks after the last immunization, C57Bl/6 mice were anesthetized with 2% Avertin and exposed to five infected mosquitoes for ~10 min. The number of mosquitoes that fed on blood was determined by observation of a red abdomen. Days four to ten post-challenge, blood smears from the tip of the mouse’s tail were collected, stained with 10% Giemsa, and examined by light microscopy to determine the presence of blood-stage parasites.

### Determination of antibody titer

To prepare the ELISA plates, 100 μL of recombinant circumsporozoite protein (rCSP) was plated at a concentration of 50 ng/mL into a 96 well MaxiSorp plate and incubated overnight at room temperature. Plates were washed three times with 1 X PBS and blocked in 1 X PBS-1% BSA (100 μL per well) for 1 h. After blocking, plates were washed three times with 1 X PBS, followed by a 1-h incubation with 100 μL of serially diluted serum samples. The initial dilution factor for the sera was 1:100 with seven serial, three-fold dilutions subsequently performed. Additionally, on each plate, antibody 2A10 was used as a positive control, and 1 X PBS-1%BSA was used as a negative control. Plates were then washed twice with 1 X PBS-0.5% Tween-20 and three times with 1 X PBS. After washing, each well was incubated with 100 μL of peroxidase-labeled goat *α*-mouse G (H+L) at a concentration of 250 ng/mL for 1 h. This was followed by three washes with 1 X PBS-0.5% Tween-20 and three washes with 1 X PBS. 100 μL of horseradish peroxidase substrate was added to each well with plates allowed to develop in the dark for 20 min. The substrate reaction was stopped by adding 50 μL of 1% SDS to each well. Each plate was read in a spectrophotometer at OD 405 nm.

### CSP antibody serum levels—2A10 equivalence

Using the collected antibody titer data, multiple serum dilutions were chosen to align with the linear range of the titration curve. For each ELISA plate, a standard curve of monoclonal antibody 2A10 was generated through three-fold titrations beginning at a concentration of 333.3 ng/mL. After determining absorbance values, GraphPad Prism was used to generate a four-parameter, non-linear regression with the 2A10 standard curve. Absorbance data from the individual serum titrations were then inputted into the standard curve and multiplied by the dilution factor to approximate the 2A10 equivalents for each serum sample. Averages of the chosen points were calculated.

### Statistical methods

To assess inter-assay and intra-variation, linear mixed effects models were used to assess the variation in the given outcome (flux or sera antibody concentration) by treatment or control group. Data were pooled across experiments, and one model was run per control/treatment group and vaccine dose (treatment groups received either two or three doses of RTS,S). The models were specified as shown in Eq. 1 below:

1 $$\begin{array}{c}{lo}{g}_{10}\left({outcome}\right)={\rm{\mu }}+{b}_{{experiment}}+\epsilon \end{array},$$

where outcome was either flux, flux reduction (log-difference with matched infected controls), or sera antibody concentrations. In the model ${\rm{\mu }}$ was the overall mean value of the outcome, each experiment had its own random intercept (denoted $b$), and the remaining residual error (intra-assay variation or measurement error) was denoted $\epsilon$. Using this model, we used the SD of the random intercepts (random effect) as an estimate of the between-experiment (inter-assay) variation. SDs of log_10_-transformed endpoints were back-transformed and interpreted as fold-change from the mean. The experiment-level means were calculated as the overall mean plus the experiment-specific random intercept (${\rm{\mu }}+{b}_{{experiment}}$) with the 95% CIs computed using a profile method implemented in the lme4 package in R^56^.

The intraclass correlation coefficient (ICC) was the proportion of between-experiment variance out of the total variance as shown in Eq. 2:

2 $${ICC}=\frac{{\sigma }_{{effect}}^{2}}{{\sigma }_{{effect}}^{2}+{\sigma }_{\epsilon }^{2}}$$

An ICC closer to 0 indicates a lower amount of variation between experiments, and therefore greater reproducibility and agreement between experiments.

To compare flux reduction between experimental groups, we fit two series of linear regression models to perform group comparisons of flux. Due to the low between-experiment variance, we pooled data across experiments rather than implement mixed effects models. The first model series was an intercept-only model for reduction in log_10_ flux relative to positive controls, with one model implemented per dose level and number of doses. In these models, an intercept significantly different from zero indicated a significant flux reduction. For the second model series, the first models were augmented to include an AB317 group covariate for direct pairwise comparisons between the given RTS,S group and the AB317 group. We compared differences in flux-reduction and calculated p-values and the fold-change in flux reduction with 95% Cis using the models. To correct for multiplicity, pairwise p-values were adjusted using Holm’s stepwise correction^57^ to control the family-wise error rate within each comparison grouping.

To model the dose-response relationship between sera antibody concentrations and flux we fit the following four parameter logistic (4PL) model with log10 flux as the outcome (y) and concentration as the input (x) as shown in Eq. 3:

3 $$y=d+\frac{a-d}{1+{(\frac{{\rm{x}}}{{\rm{EC}}50})}^{b}}.$$

In the model, $a$ is the minimum flux as sera 2A10 equivalence increases towards infinity (lower asymptote); $d$ is the maximum flux at sera 2A10 equivalence of 0 μg /mL (asymptote); EC_50_ is the concentration at which flux is 50% reduced relative to $a$ and $d$ (the point of inflection); and $b$ is the Hill slope determining steepness in the linear section of the curve. The *a* parameter was fixed at 5.37 log_10_ flux based on geometric mean of the assay background measurements and the *d* parameter was fit but determined by assuming infected controls received 0 μg of RTS,S. We fit the 4PL separately to the two- and three-administration groups (pooled over dose level) as differences in the EC_50_ provided evidence against pooling these groups. The pairwise test comparing log-transformed EC_50_ estimates between two and three administration experiments were conducted using a two-sided *t*-test (alpha = 0.05).

To model the dose-response relationship for the mosquito bite challenge assay, we used a simplified version of the 4PL model where the outcome is the probability of infection and the asymptotes were fixed to 0 and 1 (range of probabilities). There were therefore only two fitted parameters (i.e., a 2PL model): the EC_50_ (the effective concentration with 50% infection probability) and the Hill slope.

For each dose-response model, we predicted the outcome (flux or infection probability) for each dose group (total dose and dose-level) using the geometric mean concentration for the given dose-group. The means were estimated from a linear regression with log_10_-transformed sera concentration as the outcome and the dose groups as the predictors.

To determine power for future studies comparing flux reduction, we performed calculations assuming a two-sample *t*-test would be used to compare the log_10_-transformed flux with common variance. Ranges of input SDs for log_10_ flux were estimated from the minimum, mean, and maximum intra-assay variances. Statistical power for the mosquito bite challenge assay was derived using Barnard’s test for superiority (with z-pooled method)^58^ and compared test vaccine protection levels ranging from 65% to 100% to three administrations of 5 μg RTS,S/AS01 protection of 60% by sample size (*N* = 7–15; alpha = 0.05).

### Software and reproducibility

All analyses were conducted in the R programming language with the tidyverse package^59,60^. Linear mixed model fitting and regression model comparisons were conducted using the lme4 and emmeans packages^56,61^. Power calculations were performed using the pwr and Exact R packages^62^^,63^. The 4PL model was fit using the drc R package^64^.

### Reporting summary

Further information on research design is available in the Nature Research Reporting Summary linked to this article.

### Supplementary information

Establishing RTS,S/AS01 as a benchmark for comparison to next-generation malaria vaccines in a mouse model

Reporting Summary

## Data Availability

The authors confirm that the data supporting the findings of this study are available within the article and its Supplementary materials.
