# Supplementary material for: Establishing RTS,S/AS01 as a benchmark for comparison to next-generation malaria vaccines in a mouse model
Source: NPJ Vaccines. 2024 Feb 10;9:29. doi: 10.1038/s41541-024-00819-x (PMC10858969; doi:10.1038/s41541-024-00819-x)
Supplement: Supplementary file 1 — Establishing RTS,S/AS01 as a benchmark for comparison to next-generation malaria vaccines in a mouse model [file 41541_2024_819_MOESM1_ESM.pdf]

Supplementary data.

Supplementary Table 1. Adjusted flux variation estimated from using linear mixed effects models for each total dose and dose-level combination (ten models, one per row). For each animal, the adjusted log_{10} flux was calculated by subtracting the log_{10} flux from the experiment-matched mean log_{10} flux from the naïve infected control group and represents the treatment effect relative to the control group. The adjusted mean log_{10} flux (photons/sec) is the mean across experiments from the model. The intraclass correlation is the total variance attributed to differences between experiments; a value of zero implies concordance across experiments.

Number of administrations	Treatment group	Mice	Experiments	Adjusted mean flux (log10 photons/sec)	SD (log_{10} flux)		Intraclass correlation (%)
					Between-experiment	Within-experiment	
2	0.05 µg RTS,S	15	3	0.020	0.000	0.143	0.0
	0.2 µg RTS,S	15	3	0.307	0.000	0.155	0.0
	1 µg RTS,S	15	3	0.549	0.000	0.282	0.0
	5 µg RTS,S	15	3	0.716	0.085	0.221	12.9
	10 µg RTS,S	15	3	0.559	0.071	0.221	9.3
3	0.05 µg RTS,S	10	2	0.448	0.000	0.370	0.0
	0.2 µg RTS,S	10	2	0.878	0.167	0.398	14.9
	1 µg RTS,S	10	2	1.258	0.059	0.510	1.3
	5 µg RTS,S	10	2	1.277	0.000	0.361	0.0
	10 µg RTS,S	10	2	1.405	0.061	0.376	2.6

Supplementary Table 2. Statistical tests were performed using linear regression models to compare log_{10} flux reduction between each RTS,S/AS01 vaccine group and the AB317 group. All tests were two-sided (alpha = 0.05), unadjusted and adjusted (Holm correction) p-values are displayed. See Table 2 for sample sizes.

Number of administrations	Comparison	Difference in log_{10} flux reduction	p-value (unadjusted)	p-value (adjusted)
2	0.05 µg RTS,S -AB317	1.70	<0.001	<0.001
	0.2 µg RTS,S - AB317	1.41	<0.001	<0.001
	1 µg RTS,S - AB317	1.17	<0.001	<0.001
	5 µg RTS,S - AB317	1.00	<0.001	<0.001
	10 µg RTS,S - AB317	1.16	<0.001	<0.001
3	0.05 µg RTS,S -AB317	1.27	<0.001	<0.001
	0.2 µg RTS,S - AB317	0.84	<0.001	<0.001
	1 µg RTS,S - AB317	0.46	<0.001	0.002
	5 µg RTS,S - AB317	0.44	0.001	0.002
	10 µg RTS,S - AB317	0.31	0.018	0.018

Supplementary Table 3. Results from ten linear mixed effects models for the pooled treatment groups. The mean $\log_{10}$ sera 2A10 concentration (μg/mL) is the mean across experiments (the fixed effect intercept) from the model. The intraclass correlation is the total variance attributed to differences between experiments; a value of zero implies concordance across experiments.

Number of administrations		Treatment group	Mice treated	Experiments	Mean $\log_{10}$ sera 2A10 (μg/mL)	SD ($\log_{10}$ sera 2A10)		Intraclass correlation (%)
						Between-experiment	Within-experiment	
2		0.05 μg RTS,S	15	3	0.12	0.000	0.651	0.0
		0.2 μg RTS,S	15	3	1.56	0.355	0.333	53.3
		1 μg RTS,S	15	3	1.87	0.000	0.284	0.0
		5 μg RTS,S	15	3	2.02	0.000	0.176	0.0
		10 μg RTS,S	15	3	1.79	0.000	0.237	0.0
3		0.05 μg RTS,S	10	2	1.71	0.000	0.417	0.0
		0.2 μg RTS,S	10	2	2.05	0.000	0.449	0.0
		1 μg RTS,S	10	2	2.26	0.000	0.387	0.0
		5 μg RTS,S	10	2	2.32	0.000	0.157	0.0
		10 μg RTS,S	10	2	2.41	0.000	0.224	0.0

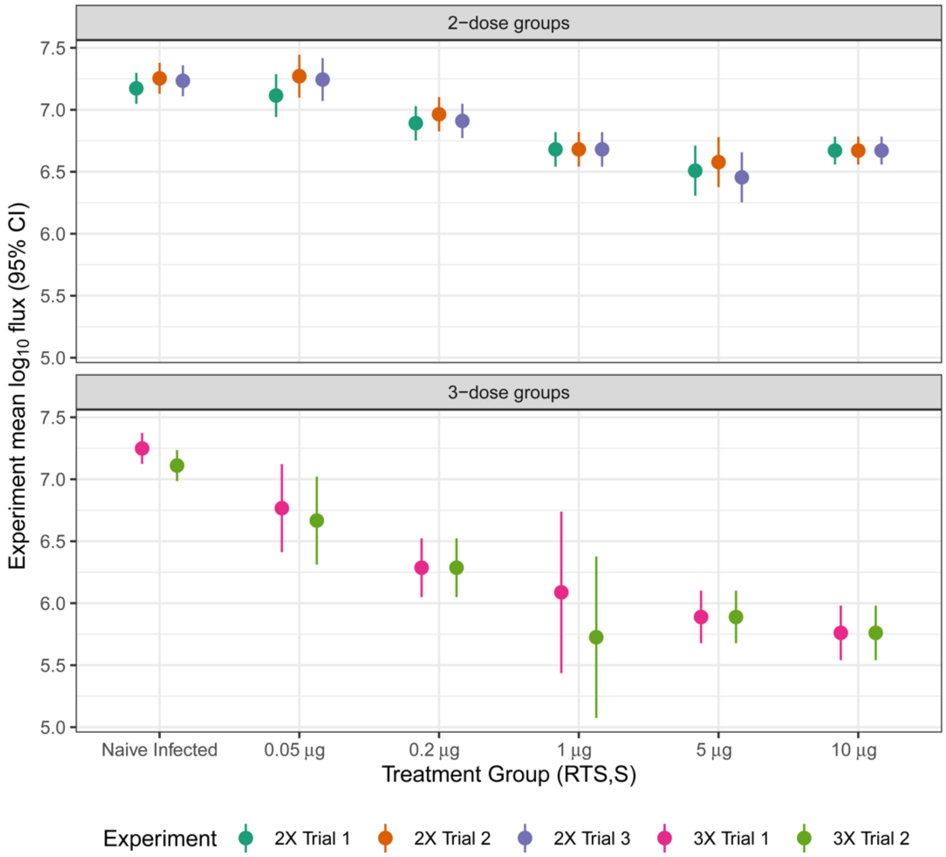

Supplementary Figure 1. Experiment-level mean log_{10} flux with 95% confidence intervals (CIs) among the vaccine groups and naïve infected controls. This was estimated using linear random effects models. CIs depict intra-assay precision while variation between the means depict inter-assay variability.

a

b

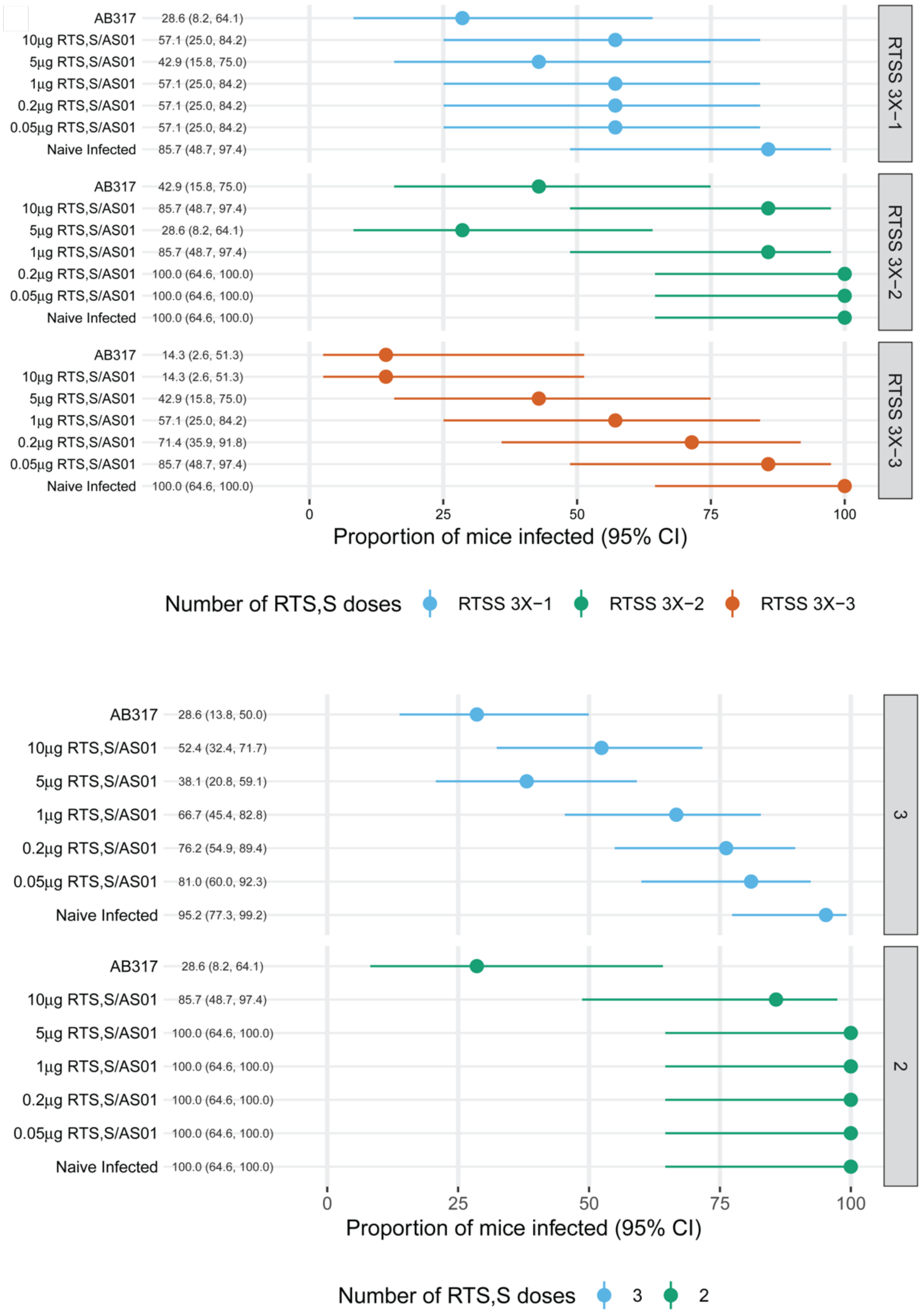

Supplementary Figure 2. **Proportion of infected mice among RTS,S/AS01 treatment and control groups with 95% Wilson Cis.** Faceted by: (a) each three-dose mosquito bite challenge experiment; and (b) number of RTS,S/AS01 doses with data pooled across the three replicate 3x vaccination experiments and the single replicate of 2x vaccination.

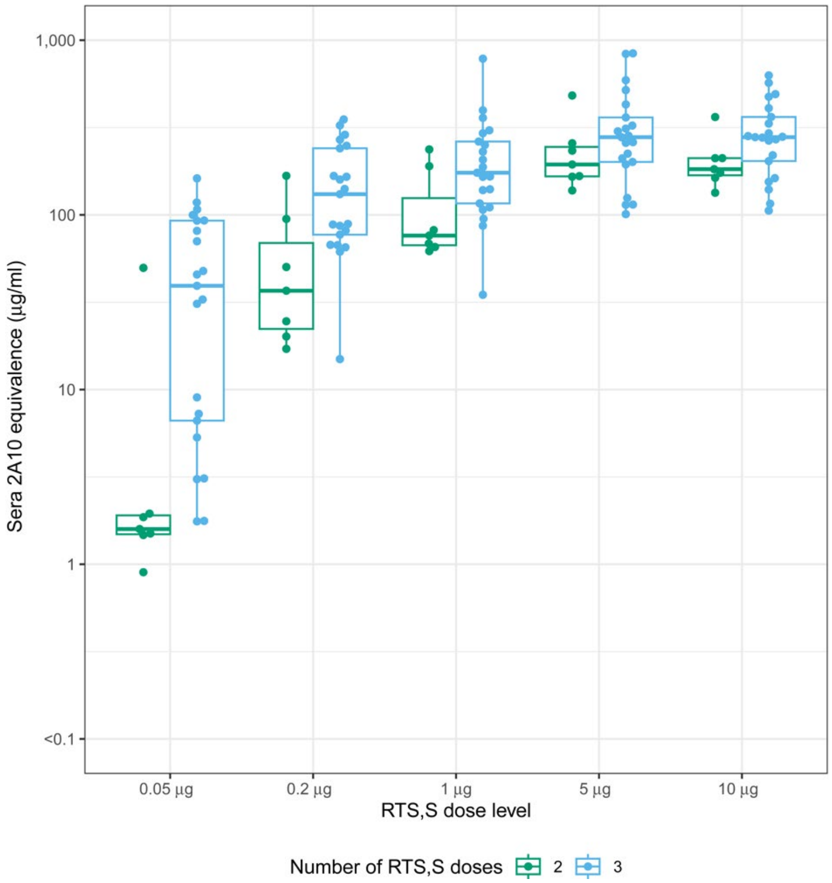

Supplementary Figure 3. Distribution of RTS,S-induced sera antibody concentrations two days prior to challenge (2A10 equivalence (µg/mL) by total doses administered and dose levels for the mosquito bite challenge experiments. Points indicate measurements from individual animals. The mid-line of the boxes denotes the median and the ends of the box denote the 25th and 75th percentiles. The whiskers denote the most extreme data points that were no more than 1.5 times the interquartile range (i.e., height of the box).

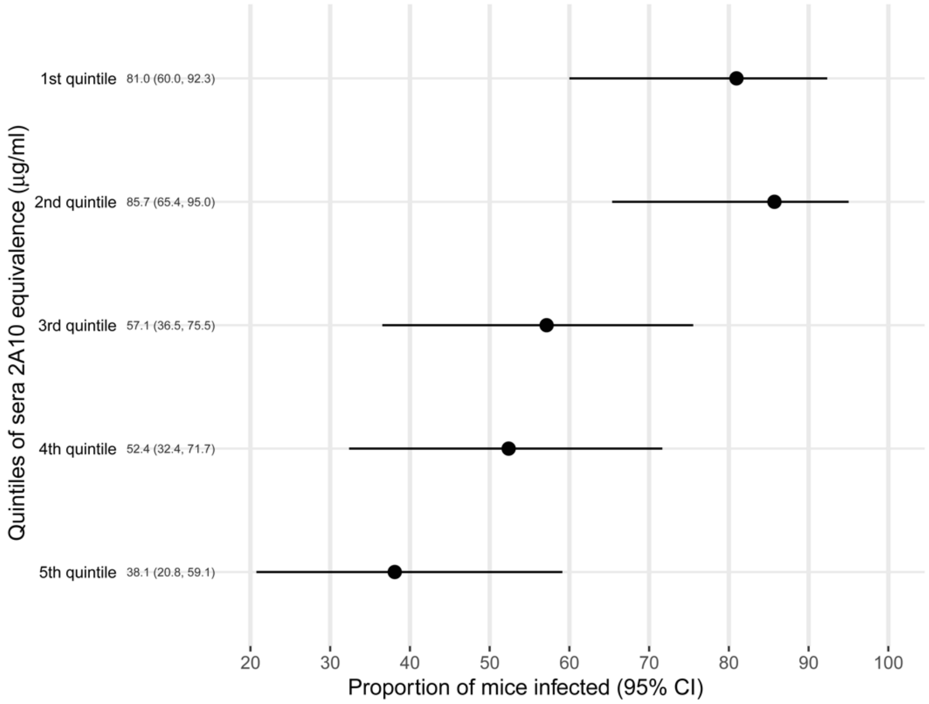

Supplementary Figure 4. Proportion of infected mice with 95% CIs (written on plot) in each quintile of antibody concentration. Antibody was measured as sera 2A10 equivalence for the 3x-dose experiments.

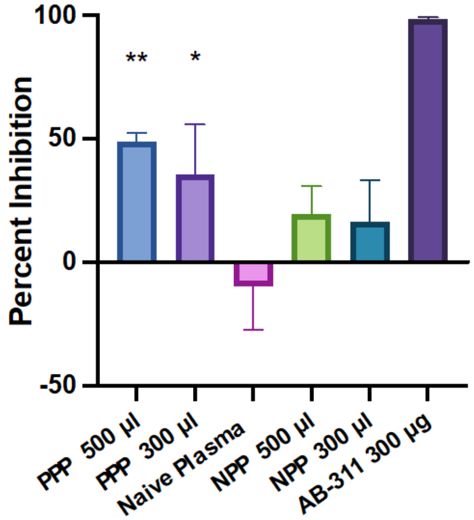

Supplementary Figure 5: Liver infection measured in mice receiving via passive transfer 300 µL or 500 µL of pooled day of challenge human plasma samples from protected (n = 24) and non-protected (n = 12) subjects. Plasma from AduFx and 2PedFx cohorts receiving the adult dose or a double pediatric dose, thus the same amount of antigen and adjuvant, on a 0, 1, 7 – month schedule of the MAL092 CHMI study. Mouse treatment groups n = 5. Plasma from pooled protected subjects conferred greater inhibition of liver infection than plasma from non-protected subjects. Asterisks (*) indicate significant reduction in liver infection compared to naïve infected mice ($p = 0.0079$ for the 500 μL cohort and 0.0317 for the 300 μL cohort, Mann-Whitney U test). Naïve infected mice and AB311 were used as negative and positive controls respectively.
