# Supplementary material for: Establishing RTS,S/AS01 as a benchmark for comparison to next-generation malaria vaccines in a mouse model
Source: NPJ Vaccines. 2024 Feb 10;9:29. doi: 10.1038/s41541-024-00819-x (PMC10858969; doi:10.1038/s41541-024-00819-x)
Supplement: Supplementary file 2 — Reporting Summary [file 41541_2024_819_MOESM2_ESM.pdf]

Corresponding author(s): DAPR #npjvaccines -03632R

Last updated by author(s): Jan 3, 2024

Reporting Summary

Nature Portfolio wishes to improve the reproducibility of the work that we publish. This form provides structure for consistency and transparency in reporting. For further information on Nature Portfolio policies, see our Editorial Policies and the Editorial Policy Checklist.

Statistics

For all statistical analyses, confirm that the following items are present in the figure legend, table legend, main text, or Methods section.

n/a	Confirmed	
☐	☒	The exact sample size (n) for each experimental group/condition, given as a discrete number and unit of measurement
☐	☒	A statement on whether measurements were taken from distinct samples or whether the same sample was measured repeatedly
☐	☒	The statistical test(s) used AND whether they are one- or two-sided<br>*Only common tests should be described solely by name; describe more complex techniques in the Methods section.*
☒	☐	A description of all covariates tested
☒	☐	A description of any assumptions or corrections, such as tests of normality and adjustment for multiple comparisons
☐	☒	A full description of the statistical parameters including central tendency (e.g. means) or other basic estimates (e.g. regression coefficient) AND variation (e.g. standard deviation) or associated estimates of uncertainty (e.g. confidence intervals)
☐	☒	For null hypothesis testing, the test statistic (e.g. F, t, r) with confidence intervals, effect sizes, degrees of freedom and P value noted<br>*Give P values as exact values whenever suitable.*
☒	☐	For Bayesian analysis, information on the choice of priors and Markov chain Monte Carlo settings
☒	☐	For hierarchical and complex designs, identification of the appropriate level for tests and full reporting of outcomes
☒	☐	Estimates of effect sizes (e.g. Cohen's d, Pearson's r), indicating how they were calculated

Our web collection on statistics for biologists contains articles on many of the points above.

Software and code

Policy information about availability of computer code

Data collection	The radiance measurements assessing liver infection were recorded by the live imager software, version 4.5.1.
Data analysis	All analyses were conducted in the R programming language with the tidyverse package. Linear mixed model fitting and regression model comparisons were conducted using the lme4 and emmeans packages. Power calculations were performed using the pwr and Exact R packages. The 4PL model was fit using the drc R package.

For manuscripts utilizing custom algorithms or software that are central to the research but not yet described in published literature, software must be made available to editors and reviewers. We strongly encourage code deposition in a community repository (e.g. GitHub). See the Nature Portfolio guidelines for submitting code & software for further information.

Data

Policy information about availability of data

All manuscripts must include a data availability statement. This statement should provide the following information, where applicable:

- Accession codes, unique identifiers, or web links for publicly available datasets
- A description of any restrictions on data availability
- For clinical datasets or third party data, please ensure that the statement adheres to our policy

The authors confirm that the data supporting the findings of this study are available within the article and its Supplementary materials.

Research involving human participants, their data, or biological material

Policy information about studies with human participants or human data. See also policy information about sex, gender (identity/presentation), and sexual orientation and race, ethnicity and racism.

Reporting on sex and gender	Plasma from healthy male and female adults participating in a malaria vaccine clinical trial (NCT03162614) in Silver Spring, MD, USA were collected and made available for this study.
Reporting on race, ethnicity, or other socially relevant groupings	Plasma from African Americans, Caucasians, and Asian Americans were used in the study.
Population characteristics	Participants in the clinical trial were male and female, comprised of African Americans, Caucasians, and Asian Americans, and mean age 31 with a range from 18 to 53.
Recruitment	Recruitment was conducted using multiple methods such as advertisements, flyers, posters, word of mouth and other institutional review board (IRB) approved methods.
Ethics oversight	WRAIR IRB was the IRB on record for the clinical study. PATH Research Determination Committee determined that the use of plasma samples from the clinical study was not human subjects research.

Note that full information on the approval of the study protocol must also be provided in the manuscript.

Field-specific reporting

Please select the one below that is the best fit for your research. If you are not sure, read the appropriate sections before making your selection.

☒ Life sciences ☐ Behavioural & social sciences ☐ Ecological, evolutionary & environmental sciences

For a reference copy of the document with all sections, see nature.com/documents/nr-reporting-summary-flat.pdf

Life sciences study design

All studies must disclose on these points even when the disclosure is negative.

Sample size	The samples size that were used for experimental was decided according to results of previous published studies (10.1186/s12936-019-3055-9; 10.1186/s12936-020-03181-0) indicating that this sample size would permit to differentiate between different experimental groups.
Data exclusions	No data was excluded for the analysis.
Replication	All experiments were repeated at least three times . All attempts of replication were successful
Randomization	Experimental groups were not randomized. In all experiments negative and positive controls were included.
Blinding	Investigators collecting data and analyzing were blinded.

Reporting for specific materials, systems and methods

We require information from authors about some types of materials, experimental systems and methods used in many studies. Here, indicate whether each material, system or method listed is relevant to your study. If you are not sure if a list item applies to your research, read the appropriate section before selecting a response.

Materials & experimental systems

n/a	Involved in the study	
☐	☒	Antibodies
☒	☐	Eukaryotic cell lines
☒	☐	Palaeontology and archaeology
☐	☒	Animals and other organisms
☒	☐	Clinical data
☒	☐	Dual use research of concern
☒	☐	Plants

Methods

n/a	Involved in the study	
☒	☐	ChIP-seq
☒	☐	Flow cytometry
☒	☐	MRI-based neuroimaging

Antibodies

Antibodies used	mAb 2A10 is a mouse mAb specific for P. falciparum CSP that recognizes the repeat domain of this antigen, the P. falciparum CSP. AB311 lot TP25172F and AB317 lot 1711017 are human immunoglobulin G1 (IgG1) mAbs were isolated from experimental clinical trial of RTS,S/AS01 MAL071 and binds to NPNA CSP repeats. They were expressed by transient transduction 0.5 L TunaCHO cultures followed by protein A purification at Lake Pharma Inc. Belmont, CA.
Validation	Characterization of performance of these mAbs in this mouse challenge model have been described elsewhere (10.1186/s12936-019-3055-9; 10.1186/s12936-020-03181-0)

Animals and other research organisms

Policy information about studies involving animals; ARRIVE guidelines recommended for reporting animal research, and Sex and Gender in Research

Laboratory animals	Female mice C57Bl/6 7-8 weeks of age were purchased from Charles River Laboratories, Frederick, MD, USA
Wild animals	This study did not involve wild animals.
Reporting on sex	These studies were performed with female mice only.<br>In previously published studies (10.1186/s12936-019-3055-9) we determined that female mice -compared to males- were more susceptible to malaria parasite infection and therefore provided a more sensitive and stringent model to evaluate mechanisms of protective immunity.
Field-collected samples	This study did not involve samples collected from the field.
Ethics oversight	All studies were approved by the Animal Care and Use Committee at Johns Hopkins University, protocol number MO18H419.

Note that full information on the approval of the study protocol must also be provided in the manuscript.

Plants

Seed stocks	*Report on the source of all seed stocks or other plant material used. If applicable, state the seed stock centre and catalogue number. If plant specimens were collected from the field, describe the collection location, date and sampling procedures.*
Novel plant genotypes	*Describe the methods by which all novel plant genotypes were produced. This includes those generated by transgenic approaches, gene editing, chemical/radiation-based mutagenesis and hybridization. For transgenic lines, describe the transformation method, the number of independent lines analyzed and the generation upon which experiments were performed. For gene-edited lines, describe the editor used, the endogenous sequence targeted for editing, the targeting guide RNA sequence (if applicable) and how the editor was applied.*
Authentication	*Describe any authentication procedures for each seed stock used or novel genotype generated. Describe any experiments used to assess the effect of a mutation and, where applicable, how potential secondary effects (e.g. second site T-DNA insertions, mosiacism, off-target gene editing) were examined.*
